# Prescription opioid use and opioid use disorder among older adults with HIV in the USA from 2008 to 2021: a retrospective repeated cross-sectional study

**DOI:** 10.1016/j.lanprc.2025.100017

**Published:** 2025-09-30

**Authors:** Stephanie Shiau, Fabrizio Drago, Carolyn W Kinkade, Kylie Getz, Greta Bushnell, Hillary Samples, Alexis A Bender, Laura Bennett, Chintan Dave, Perry N Halkitis, Tobias Gerhard, Jason A Roy, Silvia S Martins, Michael T Yin, Stephen Crystal

**Affiliations:** **Department of Biostatistics and Epidemiology** (S Shiau PhD, F Drago MPH, C W Kinkade PhD, K Getz MSc, G Bushnell PhD, L Bennett MPH, Prof P N Halkitis PhD, Prof J A Roy PhD) **and Department of Health Behavior, Society and Policy** (H Samples PhD), **Rutgers School of Public Health, New Brunswick, NJ, USA; Center for Pharmacoepidemiology and Treatment Science** (G Bushnell PhD, C Dave PharmD, Prof T Gerhard PhD) **and Center for Health Services Research** (Prof S Crystal PhD) , **Institute for Health, Health Care Policy and Aging Research, Rutgers University, New Brunswick, NJ, USA; Division of Geriatrics and Gerontology, Department of Medicine, Emory University, Atlanta, GA, USA** (A A Bender PhD); **Department of Pharmacy Practice and Administration, Ernest Mario School of Pharmacy, Rutgers University, New Brunswick, NJ, USA** (C Dave PharmD, Prof T Gerhard PhD); **Center for Health, Identity, Behavior & Prevention Studies, Rutgers School of Public Health, Newark, NJ, USA** (Prof P N Halkitis PhD); **Department of Epidemiology, Mailman School of Public Health, Columbia University Irving Medical Center, Columbia University, New York, NY, USA** (Prof S S Martins MD); **Division of Infectious Disease, Department of Medicine, Vagelos College of Physicians & Surgeons, Columbia University Irving Medical Center, New York, NY, USA** (M T Yin MD)

## Abstract

**Background:**

There is longstanding concern that people with HIV receive prescription opioids at higher rates and have a disproportionate burden of opioid use disorder (OUD) compared with their counterparts without HIV. We aimed to evaluate trends of opioid prescriptions and indicators of OUD in an understudied but growing population of older adults with HIV.

**Methods:**

For this retrospective repeated cross-sectional study, we constructed annual cohorts through a nationally representative sample of fee-for-service Medicare beneficiaries aged 65 years and older in the USA with Part D coverage (ie, prescription drug) enrolled between Jan 1, 2008, and Dec 31, 2021. Beneficiaries were eligible for inclusion in each cross-sectional cohort if they had reached the age of 65 years by Jan 1 of the calendar year and had 1 year of continuous Medicare enrolment in Part A (inpatient hospital care), B (outpatient care), and D. Beneficiaries with HIV were matched in a 1:3 ratio to beneficiaries without HIV on age, sex, race or ethnicity, US state, and dual eligibility status (Medicare and Medicaid). The main outcomes were receipt of at least one opioid prescription and any indicator of OUD (ie, formal diagnosis, medication for OUD, or opioid-related or emergency department visits) during each calendar year. Generalised estimating equations were used to estimate odds ratios (ORs) of each outcome, comparing matched beneficiaries with or without HIV. Due to data availability, our analysis of indicators of OUD was restricted to 2008–16.

**Findings:**

Across all years, 163 429 beneficiaries with HIV and 490 287 beneficiaries without HIV were included (475 516 [72·7%] were male, 178 200 [27·3%] were female; 305 776 [46·8%] were non-Hispanic White, 238 172 [36·4%] were Black [or African American], 84 128 [12·9%] were Hispanic, 8964 [1·4%] were Asian or Pacific Islander, and 16 676 [2·6%] were other races or ethnicities). During 2008–21, 57 373 (35·1%) of 163 429 people with HIV and 138 547 (28·3%) of 490 287 people without HIV received at least one opioid prescription. During 2008–16, 2408 (3·1%) of 76 637 people with HIV and 2831 (1·2%) of 229 911 people without HIV had any indicator of OUD. Across all analysed years, beneficiaries with HIV had significantly increased odds of receiving at least one opioid prescription (OR 1·38, 95% CI 1·36–1·39) and having indicators of OUD (2·61, 2·47–2·76) compared with their matched counterparts without HIV.

**Interpretation:**

Medicare beneficiaries aged 65 years and older with HIV in the USA were more likely to receive opioid prescriptions and have OUD indicators than matched beneficiaries without HIV. Our findings could help guide clinical opioid prescription guidelines and public health surveillance among this vulnerable ageing population.

**Funding:**

National Institute on Drug Abuse, New Jersey Alliance for Clinical and Translational Science, and National Center For Advancing Translational Sciences of the National Institutes of Health.

## Introduction

The availability of effective antiretroviral therapy (ART) for the treatment of HIV has increased life expectancies of people with HIV.^1^ As a result, the age distribution of people with HIV in the USA includes increasing numbers of people aged 65 years and older; this population is projected to continue to increase until 2030.^2^ Although older people with HIV have many of the same health challenges as older individuals in the general population, the effect of ageing might be more intense among people with HIV. Older people with HIV have a higher risk of comorbidities, such as neurocognitive impairment, liver disease, osteoporosis, cardiovascular disease, and frailty than individuals of a similar age without HIV.^3^ Older people with HIV also have more polypharmacy than individuals without HIV, placing them at higher risk for adverse drug–drug interactions.^4^

Chronic pain is a common and clinically significant problem experienced by up to 85% of people with HIV of all ages, attributable to multifactorial causes, such as HIV-associated and ART-associated neuropathy, and could relate to biopsychosocial risk factors such as stress, trauma, substance use, and other mental health comorbidities.^5,6^ Furthermore, opioids and non-opioids are prescribed for the management of pain for older people with HIV, with differences in patterns across global regions.^6^ Although clinical guidelines for the treatment of chronic pain state that opioids should not be a first-line treatment,^7^ there is evidence that people with HIV are more likely to receive opioid prescriptions, including at higher doses and for prolonged periods of time.^8-12^ Among patients receiving HIV care in the USA, approximately 26–40% receive opioid prescriptions,^8,11,13^ some 3–11% report misusing them,^10^ and roughly 8–17% have chronic opioid prescriptions.^14^ Medicaid claim data from 2001–09 show that the likelihood of chronic opioid use was three times higher among people with HIV than those without HIV.^9^ There is also evidence that people with HIV in the USA are more likely to receive high-risk patterns of prescriptions at high doses (≥120 mg/day) or for prolonged periods of time (≥90 days).^8,11,15^ Consequently, people with HIV are at elevated risk for opioid-related outcomes, including hospitalisations and overdose deaths.^16^ A recent US multicentre cohort study found that the prevalence of HIV was nine times higher for patients with opioid overdose presenting to the emergency department relative to the general population.^17^ Additionally, in a cross sectional study, a higher prevalence of opioid use disorders (OUDs) and other opioid-related harms was estimated among people with HIV than people without HIV.^18^

There is a paucity of information on prescription opioid use, patterns of high-risk prescription opioid use, and OUD among older people with HIV. Research into opioid prescribing and OUD among people with HIV has generally focused on adults younger than 65 years.^9,18,19^ When contextualised within the rising prevalence of opioid use and OUD in the general older population, this gap in knowledge highlights the crucial importance of research in older people with HIV, who represent a particularly high-risk group.^20^ Additionally, epidemiological studies of opioid use among people with HIV mostly focus on opioid use patterns among subgroups of people with HIV, such as people with HIV who inject drugs.^21^ Findings from these studies might not be generalisable to older people with HIV, given empirical evidence that those aged 65 years and older have different comorbidity profiles, biological changes, and risk profiles than those aged 50–64 years.^22^

There is an emerging concern that people with HIV receive prescription opioids at elevated rates and have a disproportionate burden of OUD than their counterparts without HIV. We aimed to evaluate trends of opioid prescription and indicators of OUD, including high-risk prescription patterns, among those with at least one opioid prescription and specific indicators of OUD (ie, diagnosis, use of medication for OUD [MOUD], or opioid-related hospitalisations or emergency department visits).

## Methods

### Study design and participants

In this repeated cross-sectional study, we used administrative Medicare claim data for older adults in the USA to compare outcomes for people with HIV and a matched group of older adults without HIV. In the USA, Medicare is a federal health insurance programme for people aged 65 years older and younger people with disabilities, and Medicaid is a programme for health coverage for people with limited resources. We analysed administrative claim data from listed Medicare fee-for-service beneficiaries with Part D (ie, prescription drug) coverage. Per the Centers for Medicare and Medicaid Services’ (CMS) data use polices, analyses are restricted to a maximum 20% national sample of Medicare beneficiaries, which corresponds to approximately 50% of all Part D fee-for-service beneficiaries. To meet this requirement, the dataset, obtained under a research data use agreement established with the CMS, included a 100% sample of beneficiaries residing in New Jersey and a randomly selected sample from all other US states. This design ensured adequate sample size for New Jersey-specific analyses while maintaining national representation.

We created annual cross-sectional cohorts (2008–21) of Medicare beneficiaries aged 65 years or older enrolled between Jan 1, 2008, and Dec 31, 2021 (appendix p 1). Beneficiaries were eligible for inclusion in each cross-sectional cohort if they had reached the age of 65 years by Jan 1 of the calendar year and had 1 year of continuous Medicare enrolment in Part A (inpatient care), B (outpatient care), and D (prescription drug). We excluded beneficiaries who were younger than 65 years and qualified for Medicare because of disabilities or because of endstage renal disease. We also excluded individuals enrolled in hospice care in any study year because medication recording changed across the study period.

This study used de-identified data and therefore consent was waived by the Rutgers University institutional review board. Ethical approval was obtained from the Rutgers University institutional review board (approval number MS4_Pro2020002236).

### Procedures

To identify beneficiaries living with HIV, we used the definition for HIV/AIDS from the Other Chronic Health, Mental Health, and Potentially Disabling Conditions Algorithms from the CMS Chronic Conditions Warehouse. A beneficiary met the definition of living with HIV for the year if they had at least one inpatient insurance claim for administered health-care or two non-drug claims of any other type (eg, for outpatient care or an emergency room visit) with a related HIV diagnosis occurring at least one day apart during a 2-year lookback period. Beneficiaries who did not meet this definition were considered HIV negative.

We used Master Beneficiary Summary Files from the CMS to extract sex data and race or ethnicity data. Sex was recorded as male or female. Race or ethnicity was recorded as non-Hispanic White, Black (or African American), Asian or Pacific Islander, Hispanic, American Indian or Alaskan Native, Unknown, and Other. To avoid reporting small groups, the category Other combined American Indian or Alaskan Native, Unknown, and other races and ethnicities.

We used the rural–urban commuting area codes to classify beneficiaries into metropolitan, micropolitan, small town, and rural areas based on the postal code of their residence via measures of population density, urbanisation, and daily commuting.^23^ The Elixhauser Comorbidity Index (ECI) was used to code for comorbidities, with each comorbid condition identified through ICD-9 Clinical Modification and ICD-10 Clinical Modification codes.^24^ For the ECI, we included claims from inpatient, outpatient, and carrier (ie, providers at a hospital) data files. We included 29 comorbidities from the Medicare Chronic Conditions and Other Chronic Conditions files and excluded HIV/AIDS. A beneficiary was considered to have a condition if they had a claim with the corresponding diagnosis in the given calendar year.

We estimated propensity scores with logistic regression including age, sex (female or male), race or ethnicity (non-Hispanic White, Black, Hispanic, Asian or Pacific Islander, American Indian or Alaskan Native, Other, or Unknown), US state, and dual eligibility status (ie, eligible for both Medicaid and Medicare) as predictors. We matched each beneficiary with HIV to three beneficiaries without HIV through the propensity scores by the greedy matching algorithm with a calliper width set at 0·2. Additionally, we required an exact match on age, sex, race or ethnicity, US state, and dual eligibility status. We excluded beneficiaries with HIV who had fewer than three matched counterparts without HIV for consistency in the analysis. Data on opioid prescriptions were extracted from Medicare Part D.

### Outcomes

Study outcomes and data sources are provided in the appendix (p 2). The main outcomes were receipt of one or more opioid prescriptions and any indicator of OUD during that calendar year. A composite variable representing any indicator of OUD was created by combining three opioid-related sub-indicators: diagnosis of OUD, prescription of MOUD, and opioid-related hospitalisations or emergency department visits, based on ICD-9 Clinical Modification and ICD-10 Clinical Modification codes (appendix pp 2-5). We created a count of the prevalence of each indicator in each cross-sectional year. Although we had access to Medicare Part A, B, and D data from 2008–21, we only had CMS Chronic Conditions Warehouse data for 2008–16. Since OUD was coded based on Chronic Conditions Warehouse codes, our analysis of indicators of OUD was restricted to 2008–16.

To be considered as having a prescription for MOUD, a beneficiary needed to have at least one drug claim with a US National Drug Code or at least one non-drug claim with a Health-care Common Procedure Coding System code for MOUD, both of which could include buprenorphine and oral or injectable naltrexone. Buprenorphine was exclusively coded as MOUD (appendix pp 3-5) and was included regardless of diagnosis or formulation, except for transdermal formulations that are not indicated for OUD treatment and were excluded. Methadone was covered by Medicare from 2020 onwards and coded as MOUD (appendix pp 3-5) if dispensed or administered via an opioid treatment programme.

Other outcomes were receipt of high-risk opioid prescriptions (ie, two or more overlapping prescriptions for more than 7 days; at least one period of daily morphine milligram equivalent [MME] ≥90 mg longer than 7 consecutive days; at least one period of MME ≥120 mg longer than 7 consecutive days; and long-term use, defined as ≥90 consecutive days of coverage).

Outcomes for high-risk opioid prescriptions were examined among beneficiaries that filled at least one opioid prescription.

### Statistical analysis

We analysed covariates on US census region, urbanicity, and comorbidities. US census regions were coded as northeast, midwest, south, west, and other (ie, US territories outside of the 50 US states).

We calculated descriptive statistics for beneficiary characteristics by HIV status for each calendar year. We examined differences in rural–urban commuting area and ECI in each year with χ^2^ tests. To describe trends in outcomes over time, we compared the annual prevalence (and mean annual prevalence including data from all available years) of each outcome between beneficiaries living with HIV and their matched counterparts without HIV. We used generalised estimating equation models to estimate the overall odds ratios (ORs) and 95% CIs of each outcome, comparing matched beneficiaries with and without HIV for all years combined, adjusting for calendar year, and accounting for repeated beneficiaries’ IDs and matching IDs. We used conditional logistic regression to estimate the ORs and CIs of each outcome comparing matched beneficiaries with and without HIV for each year. Regression models for our main outcomes were also stratified by sex.

We did a sensitivity analysis in which we additionally adjusted for conditions associated with both HIV and opioid use that were available in Medicare, including death during the calendar year; anxiety; depression; fibromyalgia or chronic pain or fatigue; and overall morbidity (ie, ECI). We also examined a model adjusting for individual components of the ECI, urbanicity, and age (appendix pp 19-21). As people with HIV might be less likely to be prescribed MOUD than those without HIV,^25^ we also did a sensitivity analysis excluding prescription of MOUD from the composite OUD indicator.

We considered p values less than 0·05 as significant, and all analyses were done in SAS version 8·3.

### Role of the funding source

The funders of the study had no role in study design, data collection, data analysis, data interpretation, or writing of the report.

## Results

**Figure 1:**
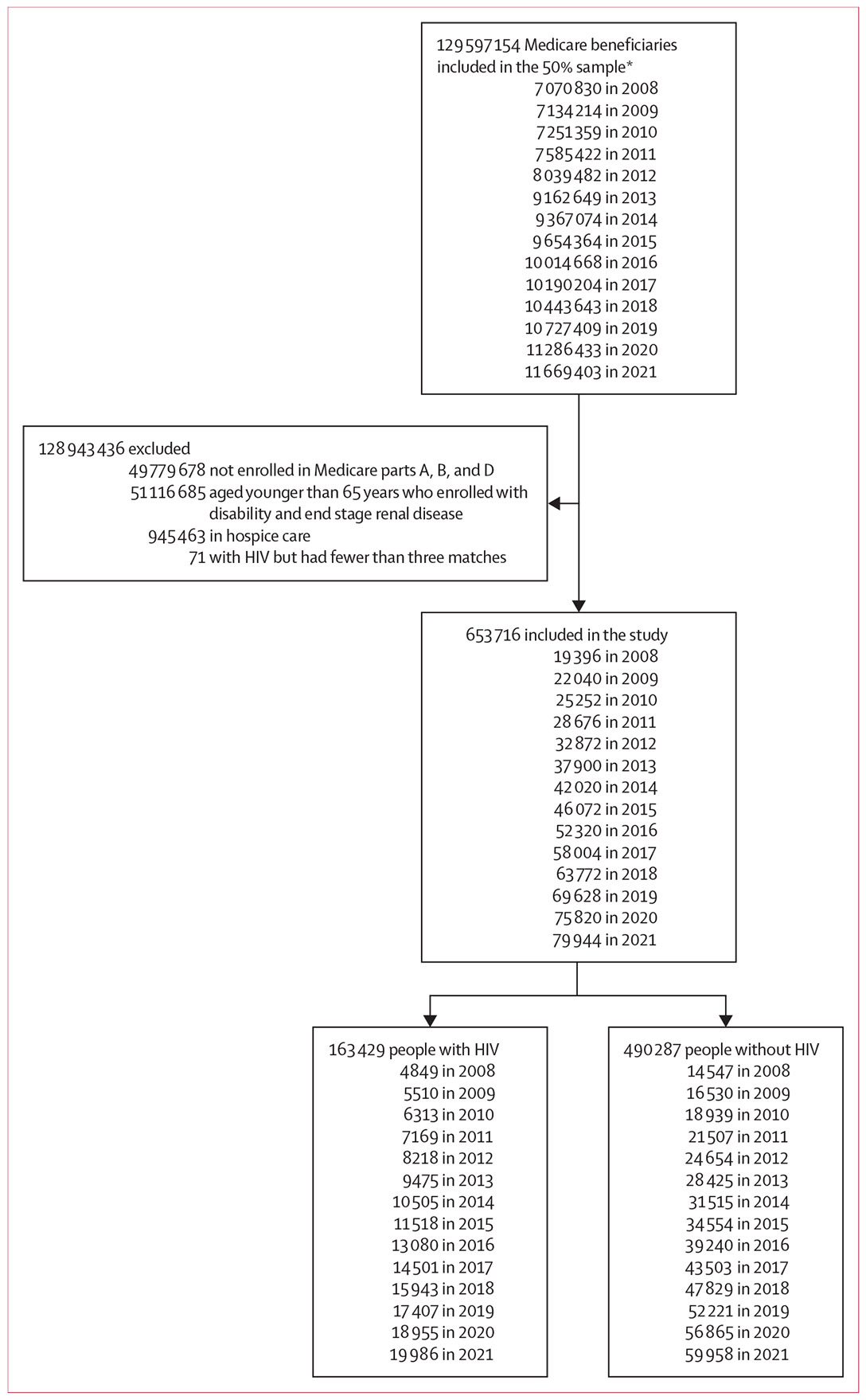
Study flow diagram *The sample was taken for Medicare fee-for-service recipients who had Part D coverage, with oversampling from New Jersey (US).

From 2008 to 2021, 653 716 Medicare beneficiaries were assessed for inclusion in the study (figure 1). Across all years, 163 429 beneficiaries with HIV were matched to 490 287 beneficiaries without HIV on age, sex, race or ethnicity, US state, and dual eligibility status (ie, Medicare and Medicaid). 71 participants with fewer than three matches were excluded (figure 1). The table shows sample characteristics of Medicare beneficiaries living with HIV and their matched beneficiaries without HIV in 2008 and 2021, with details on intervening years in the appendix (pp 6-9). The overall sample of people with HIV was predominantly male (475 516 [72·7%] were male and 178 200 [27·3%] were female), non-Hispanic White or Black (305 776 [46·8%] were non-Hispanic White, 238 172 [36·4%] were Black, 84 128 [12·9%] were Hispanic, 8964 [1·4%] were Asian or Pacific Islander, and 16 676 [2·6%] were Other), and aged 65–69 years (304 266 [46·5%]; table; appendix pp 6-9). Of the US census regions, the greatest proportion of people with HIV resided in the south, followed by the northeast (table; appendix pp 6-9). Most people with HIV lived in metropolitan areas, and the proportion living in metropolitan areas was significantly greater than beneficiaries without HIV in each calendar year (data not shown). Beneficiaries living with HIV had significantly higher ECI values (mean 3·4 [SD 2·6] in 2008; 4·6 [3·5] in 2021) than people without HIV (2·9 [2·4] in 2008; 4·0 [3·3] in 2021; p<0·0001). In all years, the largest difference in ECI by HIV status was among people with at least six comorbidities (appendix pp 6-9). The proportion of individuals with multiple (ie, ≥6) diagnosed comorbidities increased during the study period for both people with HIV (from 982 [20·3%] of 4849 in 2008 to 6557 [32·8%] of 19 986 in 2021) and without HIV (from 2022 [13·9%] of 14 547 in 2008 to 15 986 [26·7%] of 59 958 in 2021; appendix pp 6-9).

**Figure 2:**
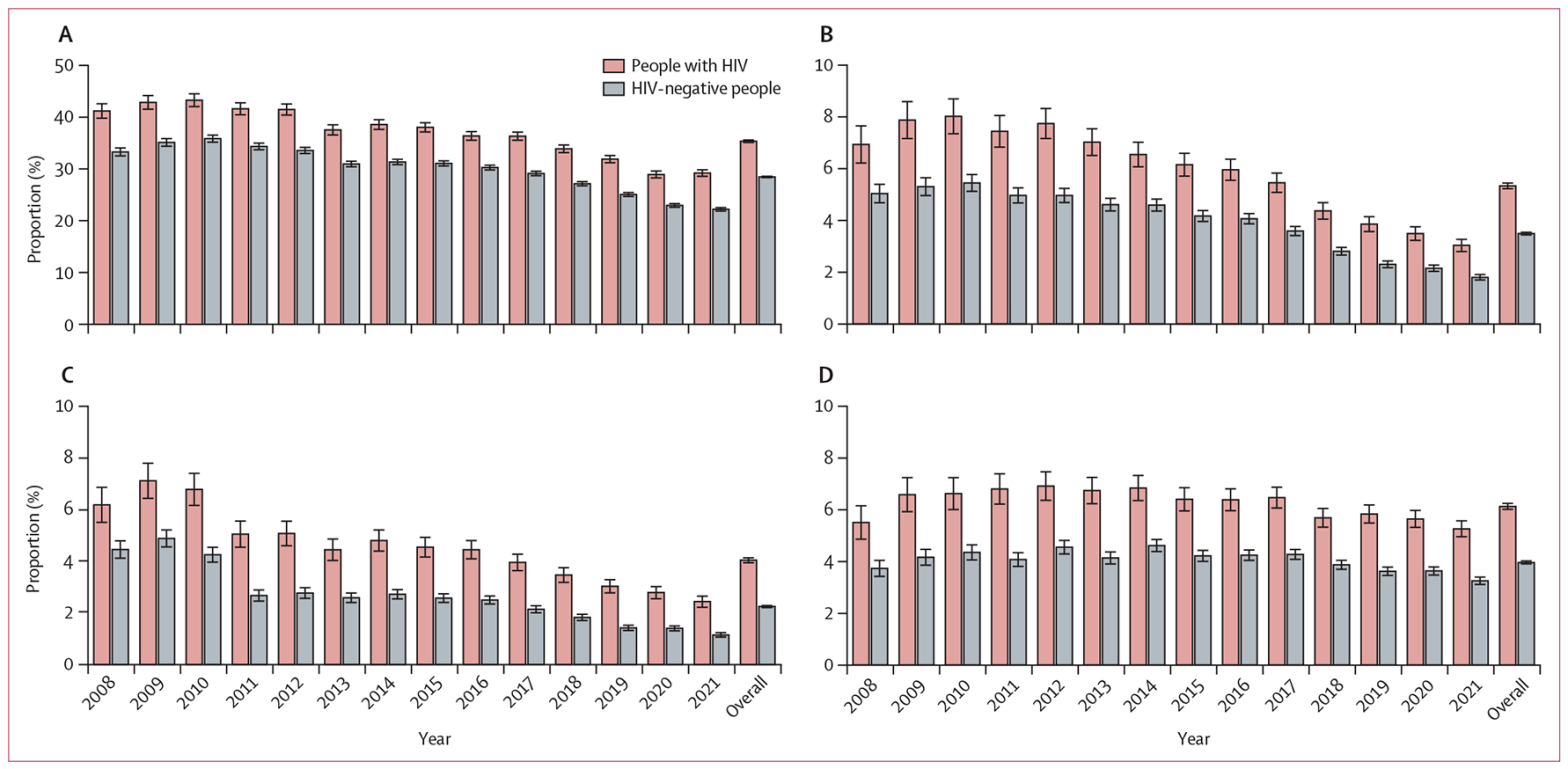
Proportion of Medicare beneficiaries with or without HIV receiving opioid prescriptions during 2008–21 Data are the proportion of beneficiaries receiving at least one opioid prescription (A), receiving at least two overlapping prescriptions for longer than 7 days (B), with at least one incident of total daily MMEs of at least 90 mg for longer than 7 consecutive days (C), or with at least 90 consecutive days of opioid coverage (D). Note that the y axis in panel A is on a different scale. Error bars are 95% CI. The proportion of beneficiaries with at least one incident of total daily MMEs of at least 120 mg for longer than 7 consecutive days is in the appendix (pp 11-12). MME=morphine milligram equivalent.

From 2008 to 2021, 57 373 (35·1%) of 163 429 people with HIV and 138 547 (28·3%) of 490 287 people without HIV received at least one opioid prescription during each calendar year. Across all years, a higher proportion of beneficiaries living with HIV received at least one opioid prescription during the calendar year than beneficiaries without HIV (figure 2; appendix p 10). High-risk opioid prescriptions were also more prevalent in beneficiaries living with HIV, with a higher percentage of people with HIV receiving overlapping opioid prescriptions for more than 7 consecutive days (people with HIV 8658 [5·3%] *vs* people without HIV 17 031 [3·5%]), having a period of total daily MMEs greater than 90 mg (people with HIV 6579 [4·0%] *vs* people without HIV 10 975 [2·2%]) and greater than 120 mg (people with HIV 5001 [3·1%] *vs* people without HIV 7579 [1·6%]) for more than 7 consecutive days, and with an opioid coverage longer than 90 consecutive days (people with HIV 9941 [6·1%] *vs* people without HIV 19 280 [3·9%]).

During 2008–16, indicators of OUD were similarly higher in beneficiaries living with HIV than their matched counterparts without HIV (people with HIV 2408 [3·1%] *vs* people without HIV 2831 [1·2%]; appendix pp 1, 10). Additionally, use of MOUD and opioid-related hospitalisations or emergency department visits were higher in people with HIV than in people without HIV (appendix p 10). These findings were consistent in all individual years; however, the number of people with HIV with an indicator of OUD increased from 101 (2·1%) in 2008 to 613 (4·7%) in 2016 (appendix pp 13-14). Across all individual analysed years, of people who had at least one indicator of OUD, most people with HIV reported one indicator (25·7–42·7%) or two or more indicators (57·3–74·3%; appendix pp 13-15).

**Figure 3:**
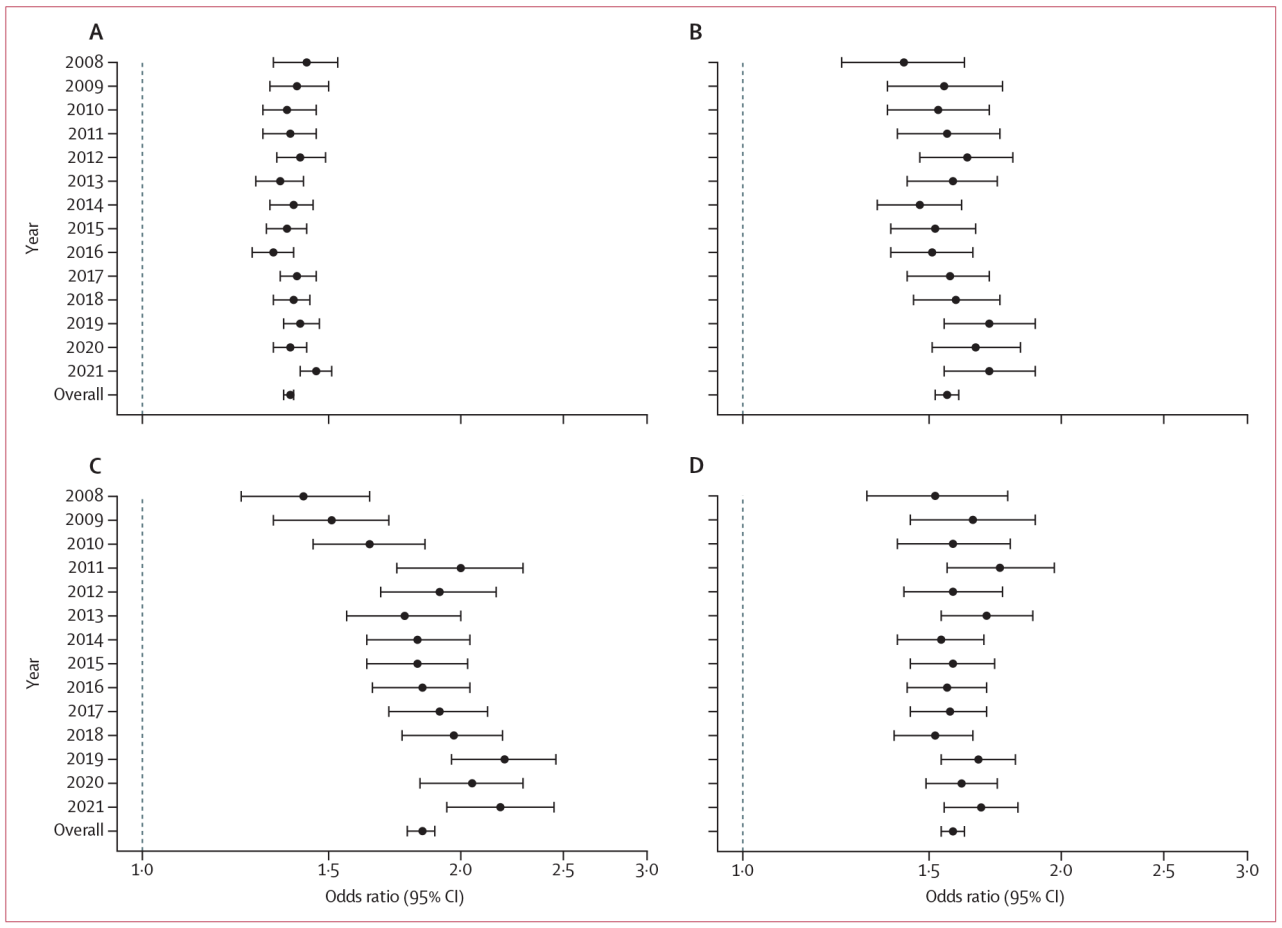
Odds ratios of opioid prescriptions in Medicare beneficiaries with HIV compared with matched beneficiaries without HIV during 2008–21 Data are odds ratio (95% CI) for beneficiaries receiving at least one opioid prescription (A), receiving at least two overlapping prescriptions for longer than 7 days (B), with at least one incident of total daily MMEs of at least 90 mg for longer than 7 consecutive days (C), or with at least 90 consecutive days of opioid coverage (D). The proportion of beneficiaries with at least one incident of total daily MMEs of at least 120 mg for longer than 7 consecutive days is in the appendix (pp 11-12). MME=morphine milligram equivalent.

**Figure 4:**
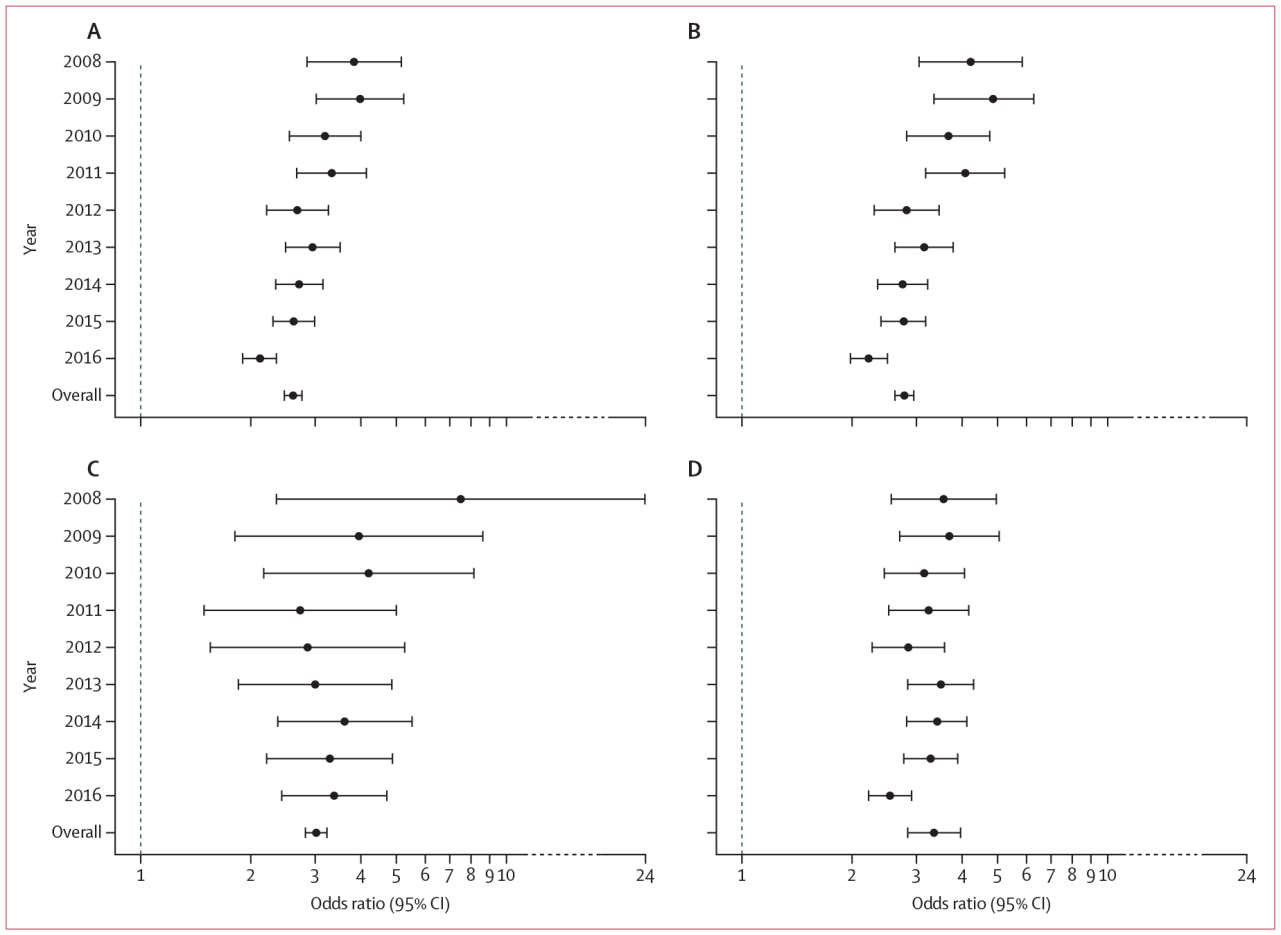
Odds ratios of indicators of OUD in Medicare beneficiaries with HIV compared with matched beneficiaries without HIV during 2008–16 Data are odds ratio (95% CI) for beneficiaries with indicators of OUD during that calendar year (A), with diagnosis of OUD (B), with use of medication for OUD (C), or with opioid-related hospitalisations or emergency department visits (D). OUD=opioid use disorder.

Beneficiaries with HIV had significantly increased odds of receiving at least one opioid prescription (overall OR 1·38, 95% CI 1·36–1·39) and having indicators of OUD (2·61, 2·47–2·76) than their matched counterparts without HIV. This finding was consistent in all individual years (figure 3; figure 4; appendix pp 16-18). However, the ORs of people with HIV having an OUD indicator decreased from 3·83 (95% CI 2·85–5·16) in 2008 to 2·12 (1·90–2·35) in 2016. ORs of high-dose opioid prescriptions (total daily MME ≥120 mg) increased from 1·53 (95% CI 1·28–1·82) in 2008 to 2·46 (2·15–2·82) in 2021 for people with HIV compared to people without HIV.

We also stratified findings by sex and found similar patterns between sexes (appendix pp 19-20). Similar findings were observed for receipt of at least two overlapping prescriptions for more than 7 consecutive days (OR 1·56, 95% CI 1·52–1·60), at least 90 mg (1·84, 1·78–1·89) and at least 120 mg (2·01, 1·94–2·09) total MME daily dose, at least 90 consecutive days of coverage (1·58, 1·54–1·62), clinical diagnosis of OUD (2·78, 2·62–2·95), use of MOUD (3·35, 2·84–3·95), and opioid-related hospitalisations or emergency department visits (3·02, 2·82–3·23). Findings were again consistent in individual years (figure 3; figure 4; appendix pp 16-18).

Sensitivity analyses using separate models adjusted for death during the cross-sectional year; anxiety; depression; fibromyalgia or chronic pain or fatigue; and overall morbidity (ie, ECI) had similar findings, albeit with slightly weaker associations (appendix pp 21-23), as did sensitivity analyses excluding prescription of MOUD from the composite OUD indicator (appendix pp 10, 13-14, 21-23).

## Discussion

In this retrospective repeated cross-sectional study, we found that older people with HIV enrolled in Medicare in the USA were more likely to receive opioid prescriptions and have indicators of OUD than individuals without HIV. Despite a lower proportion of beneficiaries (ie, both people with HIV and people without HIV) receiving an opioid prescription in 2021 than in 2008, we found consistent increased likelihoods of opioid prescription in people with HIV compared with individuals without HIV across the years analysed. ORs of people with HIV having an OUD indicator decreased from 3·83 (95% CI 2·85–5·16) in 2008 to 2·12 (1·90–2·35) in 2016, and ORs of high-dose opioid prescriptions (total daily MME ≥120 mg) increased from 1·53 (95% CI 1·28–1·82) in 2008 to 2·46 (2·15–2·82) in 2021 for people with HIV compared to people without HIV. People with HIV were also at greater risk of receiving overlapping and long-term prescriptions. ORs of clinical OUD diagnosis, opioid-related hospitalisation and emergency department visits, and use of MOUD were higher for people with HIV than people without HIV but declined from 2008 to 2016. Adjustment for death during the cross-sectional year; anxiety; depression; fibromyalgia or chronic pain or fatigue; and overall morbidity did not substantially change results, suggesting that increased opioid use persists in older people with HIV, despite adjustment for medical burden. The study period reflects a transition period in the medical community’s understanding of the potential harms of opioids and decreasing opioid prescriptions; yet, given an increase in the study-eligible population from 2008 to 2016, there were more people with HIV with any indicator of OUD in 2016 than 2008.

Studies of opioid prescriptions in people with HIV in the USA have consistently reported higher rates of opioid prescriptions and indicators of OUD than the general population.^8,10-12^ Generally, most of these studies have focused primarily or exclusively on people with HIV younger than 65 years.^18,19^ Medicaid claim data from adult beneficiaries (aged 18–65 years) between 2001 and 2009 showed that the likelihood of chronic opioid use was three times higher among people with HIV than those without HIV.^9^ In previous research of patients receiving HIV care in the USA, 17–40% received opioid prescriptions,^8,11,13,14,26^ and we found that in older adults these percentages were 22–43%. Earlier research of people with HIV indicated that at least 3% report one or more indicators of OUD;^10^ in this study of older people with HIV, 2–5% had indicators of OUD (appendix p 13). We found that 5–7% of older people with HIV had long-term opioid prescription (ie, ≥90 consecutive days), whereas this was previously reported as 8–17% of people with HIV of all ages.^14^ In the current study of older adults, people with HIV were on average 3·1 times more likely to have an indicator of OUD than individuals without HIV, indicating that OUD might be greater in an ageing population with HIV than in those without.

To summarise, we found that opioid prescriptions and indicators of OUD in older people with HIV were similar to earlier literature in younger populations, emphasising the importance of interventions that target older people with HIV. Furthermore, our matched study design allowed for the direct comparison of opioid use and indicators of OUD in older adults with HIV versus those without HIV.

In the USA, the total number of opioid prescriptions dispensed peaked in 2010 and declined thereafter, as public concern about the negative effects of opioids increased and state and federal policies designed to curb opioid overuse were implemented.^27^ In this study, we found that receipt of long-term (≥90 days) opioid prescriptions among people with HIV did not necessarily follow these decreasing trends—ie, the proportion of people with HIV and without HIV who had at least one opioid prescription and received long-term prescriptions was stable from 2008 to 2021. Additionally, the proportion of participants with any indicator of OUD increased from 2008 to 2016, driven by OUD diagnosis and opioid-related hospitalisations or emergency department visits, with smaller increases in MOUD. Future studies are warranted to better understand whether the observed increases in MOUD could be due to increasing access to treatment or use of buprenorphine for pain management. In addition, the higher odds of MOUD receipt for older people with HIV than those without HIV were in contrast to Tsui and colleagues’ retrospective cohort study,^25^ which found less documented MOUD for adults with HIV of all ages. To ensure our findings were robust, we did a sensitivity analysis excluding prescription of MOUD from the composite OUD indicator and had similar findings.

The potential harm of opioid use in this population of older people with HIV is multifaceted. Opioid prescriptions are known to be associated with overdose and OUD.^28^ Opioid use and OUD can lead to adverse health outcomes including virological failure^13,29^ and non-HIV morbidity and mortality.^28^ There is also the potential for increased falls and pneumonia, which are more prevalent with opioid use.^30,31^ However, little is understood about the association between prescribed opioids and the risk of cardiovascular disease. A recent study from the US Veterans Aging Cohort Study, a national prospective cohort of veterans with and without HIV, found that those receiving opioids had an increased hazard of incident cardiovascular disease than those not receiving opioids.^32^ It is important for future research to study these outcomes in older adults with HIV, as they face increased risk from prescription opioid use compounded by age-related factors. The US Centers for Disease Control and Prevention has warned that older adults face increased risk from prescription opioid use, due to factors such as increased potential for drug–drug interactions, increased rates of cognitive impairment leading to dosing errors, and decreased renal function that reduces the threshold for overdose.^33^ These risks should also be considered given the polypharmacy and multiple substance use patterns of older people with HIV.^34^ Future research should assess any associations between prescription opioid use and non-opioid related morbidity. Finally, there is also the risk of drug interactions between ART and opioids. In-vitro studies have shown numerous interactions between opioids and HIV, such as shortened time to develop viral resistance to ART.^35^ Given the rapidly increasing number of older people with HIV, and the risks older people with HIV might face from prescription opioid use, more research is needed on opioid-related morbidity among people with HIV aged 65 years and older.

Our study has several limitations. The use of Medicare claim data from an exclusively US population limits the generalisability of these findings across more diverse populations and countries with dissimilar health-care systems. Internationally, the USA has the highest opioid prescribing rates of all countries, and our observed findings would not be present in settings that rely on non-opioid treatments for chronic pain.^36^ Moreover, we required individuals to have continuous enrolment in Medicare Parts A, B, and D for each year, not for the entire study period, affecting who could be included in the study population and potentially leading to bias, as people with HIV might have additional reasons or resources to avoid lapses in enrolment. We excluded beneficiaries in hospice care because prescriptions were not coded during part of the years examined, which reduces the generalisability of this research to those in hospice care. We excluded beneficiaries with HIV who had less than three matched counterparts without HIV; this could introduce selection bias against the oldest people with HIV (eg, ≥80 years) who would be less likely to have three matches, as well as people of minoritised race or ethnicity, such as American Indian or Alaskan Natives. We did not exclude individuals with a cancer diagnosis because a CMS study found only a small difference in the prevalence of opioid prescriptions between samples that included or excluded beneficiaries with cancer diagnoses.^37^ In terms of the exposure, some people who are categorised as opioid naive are people who have never filled any prescription and could represent a part of the population with poor health-care access. As this study focuses on people with HIV aged 65 years and older, we are unable to comment on the continuity of opioid prescriptions from younger ages to older ages, or whether there are incident opioid prescriptions in older people with HIV. Additionally, medication use was determined with Part D data and prescription information; however, we do not know whether the individual took the medication or if they obtained medication outside the Medicare system. In the general population, research indicates that many individuals dealing with noncancer pain limit the use of their opioid prescription.^38^ Our study also did not include analysis of opioid deaths as that was not the focus of the research. Although one of the strengths of this study is the use of Medicare data, which is a rich and comprehensive source, there are drawbacks relevant to missing data and misclassification. It is possible that diagnoses are incomplete and less rigorous than accessing data from the full medical record of an individual. For example, the criteria we used to identify people with HIV (at least one inpatient claim or two claims of another type) could have resulted in classifying people with HIV as those without HIV. The result of this type of misclassification would bias the results towards the null. Misclassification of included demographic information (eg, race or ethnicity) is also a possibility. Due to not having the necessary information, we were unable to examine the role of gender or socioeconomic status (aside from dual eligibility status) in our analyses. Finally, we did not have information on the prescriber’s training and knowledge of opioid risks, which could influence patient outcomes.

Although guidelines emphasise the primary role of non-pharmacological and non-opioid pharmacological interventions to promote safe and effective chronic pain management, we found that older people with HIV in the USA are more likely to have received opioid prescriptions, at higher doses and for longer periods, and are more likely to have indicators of OUD than the general population. Opioid prescribing remains common despite known opioid-related harms, and the risk of drug interactions and non-overdose morbidity remains understudied in older people with HIV. This study provides more information for practitioners to consider in their clinical decisions for older people with HIV and chronic pain. Public health researchers and clinicians could consider alternative therapies for pain as well as screening and prevention programmes for OUD in older adults living with HIV. Future research is warranted on the interaction of ART and OUD.

## Supplementary Material

Supplementary appendix

## Figures and Tables

**Table:** Characteristics of included Medicare beneficiaries by HIV status and year

	2008		2021	
	People with HIV (n=4849)	People without HIV (n=14 547)	People with HIV (n=19 986)	People without HIV (n=59 958)
Sex				
Female	1530 (31·6%)	4590 (31·6%)	4920 (24·6%)	14760 (24·6%)
Male	3319 (68·4%)	9957 (68·4%)	15066 (75·4%)	45198 (75·4%)
Age, years	71·2 (5·3)	71·2 (5·3)	71·9 (5·2)	71·9 (5·2)
Age group, years				
65–69	2348 (48·4%)	7044 (48·4%)	8058 (40·3%)	24179 (40·3%)
70–74	1487 (30·7%)	4458 (30·6%)	7108 (35·6%)	21317 (35·6%)
75–79	611 (12·6%)	1837 (12·6%)	2996 (15·0%)	8987 (15·0%)
≥80	403 (8·3%)	1208 (8·3%)	1824 (9·1%)	5473 (9·1%)
Race or ethnicity*				
White	1977 (40·8%)	5931 (40·8%)	10690 (53·5%)	32070 (53·5%)
Black	1984 (40·9%)	5952 (40·9%)	6166 (30·9%)	18498 (30·9%)
Hispanic	761 (15·7%)	2283 (15·7%)	2147 (10·7%)	6441 (10·7%)
Asian or Pacific Islander	75 (1·5%)	225 (1·5%)	302 (1·5%)	906 (1·5%)
Other	52 (1·1%)	156 (1·1%)	681 (3·4%)	2043 (3·4%)
Dual eligibility status†	3037 (62·6%)	9111 (62·6%)	8981 (44·9%)	26943 (44·9%)
US census region‡				
Northeast	1475 (30·4%)	4425 (30·4%)	5551 (27·8%)	16653 (27·8%)
Midwest	493 (10·2%)	1479 (10·2%)	2231 (11·2%)	6693 (11·2%)
South	2051 (42·3%)	6153 (42·3%)	7762 (38·8%)	23286 (38·8%)
West	812 (16·7%)	2436 (16·7%)	4404 (22·0%)	13212 (22·0%)
Other	18 (0·4%)	54 (0·4%)	38 (0·2%)	114 (0·2%)
Rural–urban community area				
Metropolitan	4445 (91·7%)	12319 (84·7%)	18050 (90·3%)	50272 (83·8%)
Micropolitan	241 (5·0%)	1180 (8·1%)	1104 (5·5%)	5155 (8·6%)
Small town	96 (2·0%)	622 (4·3%)	464 (2·3%)	2684 (4·5%)
Rural	59 (1·2%)	404 (2·8%)	350 (1·8%)	1792 (3·0%)
ECI score§	3·4 (2·6)	2·9 (2·4)	4·6 (3·5)	4·0 (3·3)
ECI score group				
0	512 (10·6%)	2214 (15·2%)	1311 (6·6%)	7693 (12·8%)
1	805 (16·6%)	2499 (17·2%)	2455 (12·3%)	7521 (12·5%)
2	832 (17·2%)	2767 (19·0%)	2833 (14·2%)	8621 (14·4%)
3	679 (14·0%)	2282 (15·7%)	2654 (13·3%)	8074 (13·5%)
4–5	1039 (21·4%)	2763 (19·0%)	4176 (20·9%)	12063 (20·1%)
≥6	982 (20·3%)	2022 (13·9%)	6557 (32·8%)	15986 (26·7%)
Death during index year	485 (10·0%)	744 (5·1%)	1285 (6·4%)	2990 (5·0%)

Data are n (%) or mean (SD). Beneficiaries were matched on age, sex, race or ethnicity, US state, and dual eligibility status. A characteristics table for all years from 2008–21 is in the appendix (pp 6-9). *Race or ethnicity data were collected from Centers for Medicare and Medicaid Services beneficiary data; the category Other represents American Indian, Alaskan Native, Unknown, and other races and ethnicities. †Dual eligibility status means an individual was eligible for both Medicaid and Medicare. ‡The category Other of US census region refers to territories outside of the 50 US states. §The ECI was used to code for comorbidities, with each comorbid condition being identified with the ICD-9 Clinical Modification and ICD-10 Clinical Modification codes. ECI=Elixhauser Comorbidity Index.

## Data Availability

The data use agreement of the proprietary data used for this study does not allow us to submit or share the data used for our analyses. This study was completed in part by research services and/or survey/data resources provided by the Institute for Health Survey/Data Core at Rutgers University.

## Research in context

### Evidence before this study

We searched PubMed using the search terms “opioid”, “opioid use”, “opioid use disorder”, and “HIV” for literature published between Jan 1, 2010, and Sept 1, 2024 in English. We identified literature examining opioid use among people living with HIV, although no studies to date have reported exclusively on older adults. A US cross-sectional study concluded that opioid prescriptions were more likely among people with HIV than among matched controls. An analysis of Medicaid pharmacy claims showed that people with HIV received roughly twice as many opioid prescriptions as people without HIV. A US multi-site longitudinal observational study found that a large proportion of people with HIV received opioid prescriptions and noted that prescription patterns associated with risk of opioid use disorder (OUD; ie, high dose prescriptions, refills, and multiple prescriptions or prescribers) were common. These trends are concerning as opioid misuse has been associated with negative outcomes in the USA, including lower antiretroviral therapy (ART) adherence and viral suppression and higher risk of HIV transmission to sexual partners. Conversely, despite risks of long-term opioid therapy, research in a national clinic-based cohort in the USA found that long-term opioid therapy was associated with lower risk of losing viral suppression once it was achieved. ART has a positive effect on the lifespan of people with HIV and, for the first time, a large proportion of people are encountering health challenges associated with HIV compounded by age-related health decline. Currently, although increased use of opioids and OUD in people with HIV is established, public health authorities are only beginning to understand the health landscape of older people with HIV, many of whom have chronic pain and age-related health conditions and might be prescribed opioids.

### Added value of this study

To our knowledge, this study is the first to examine opioid use and indicators of OUD specifically among older (≥65 years) adults with HIV. We found that older US federal health insurance beneficiaries with HIV had an increased likelihood of receiving an opioid prescription compared with those without HIV. Additionally, older people with HIV were more likely to receive high-risk prescriptions (ie, higher dose, overlapping prescriptions, and for a longer duration). Similar to studies in adults with HIV of all ages, we found a higher prevalence of OUD indicators (ie, diagnosis, hospitalisation, and medication for OUD) for beneficiaries with HIV than beneficiaries without HIV.

### Implications of all the available evidence

This study extends previous knowledge of opioid effects among people with HIV, to highlight the previously unaddressed additional risks faced by older adults (ie, more likely to be prescribed opioids and have indicators of OUD). As there is an ongoing shift in the age of the population affected by HIV, this study highlights potential adverse outcomes of opioid use for the treatment of chronic pain in this population. In light of this study’s finding, clinicians treating older adults with HIV should consider alternative therapies for pain, and public health researchers and policy makers should consider screening and prevention programmes for OUD in older adults living with HIV. Future research should explore the interaction of ART and OUD, as well as morbidity and mortality associated with opioid use in older people with HIV.
