# Supplementary material for: Prescription opioid use and opioid use disorder among older adults with HIV in the USA from 2008 to 2021: a retrospective repeated cross-sectional study
Source: Lancet Prim Care. Author manuscript; Available in PMC 2025 Dec 18. (PMC12709593; doi:10.1016/j.lanprc.2025.100017)
Supplement: Supplementary appendix [file NIHMS2124829-supplement-Supplementary_appendix.pdf]

THE LANCET
Primary Care

Supplementary appendix

This appendix formed part of the original submission and has been peer reviewed.
We post it as supplied by the authors.

Supplement to: Shiau S, Drago F, Kinkade CW, et al. Prescription opioid use and opioid use disorder among older adults with HIV in the USA from 2008 to 2021: a retrospective repeated cross-sectional study. *Lancet Prim Care* 2025. https://doi.org/10.1016/j.lanprc.2025.100017

<u>Appendix Table of Contents</u>

p. 1: **Supplementary Figure 1.** Percentage of Medicare beneficiaries with HIV and matched HIV-negative beneficiaries with opioid use disorder outcomes, by calendar year

p. 2: **Supplementary Table 1.** Medicare Part D and Chronic Conditions codes and associated study outcomes

pp. 3-5: **Supplementary Table 2.** Coding rubric for opioid use disorder outcomes

pp. 6-9: **Supplementary Table 3.** Characteristics of beneficiaries included in the analysis, by HIV status and year (2008-2021)

p. 10: **Supplementary Table 4.** Average annual prevalence and overall odds ratios (OR) and 95% confidence interval (95% CI) of opioid prescription measures and indicators of OUD in Medicare beneficiaries with HIV compared to HIV-negative beneficiaries for all years combined (2008-2021)

pp. 11-12: **Supplementary Table 5**. Percentage of Medicare beneficiaries with HIV and matched HIV-negative beneficiaries receiving one or more opioid prescription, by calendar year

p. 13: **Supplementary Table 6.** Percentage of Medicare beneficiaries with HIV and matched HIV-negative beneficiaries with opioid use disorder outcomes, by calendar year

pp. 14-16: **Supplementary Table 7.** Odds ratios of opioid prescription measures in Medicare beneficiaries with HIV compared to matched HIV-negative beneficiaries, unadjusted and adjusted for anxiety, fibromyalgia/chronic pain/fatigue, depression, or ECI, by calendar year

p. 17: **Supplementary Table 8.** Odds ratios of opioid prescriptions in PWH compared to HIV-negative stratified by sex (unadjusted), by calendar year

p. 18: **Supplementary Table 9.** Odds ratios of any opioid use disorder (OUD) indicator in PWH compared to HIV-negative stratified by sex (unadjusted), by calendar year

pp. 19-21: **Supplementary Table 10.** Odds ratios of indicators of OUD in Medicare beneficiaries with HIV compared to matched HIV-negative beneficiaries, unadjusted and adjusted for anxiety, fibromyalgia/chronic pain/fatigue, depression, or ECI, by calendar year

Supplementary Figure 1. Percentage of Medicare beneficiaries with HIV and matched HIV-negative beneficiaries with opioid use disorder outcomes, by calendar year

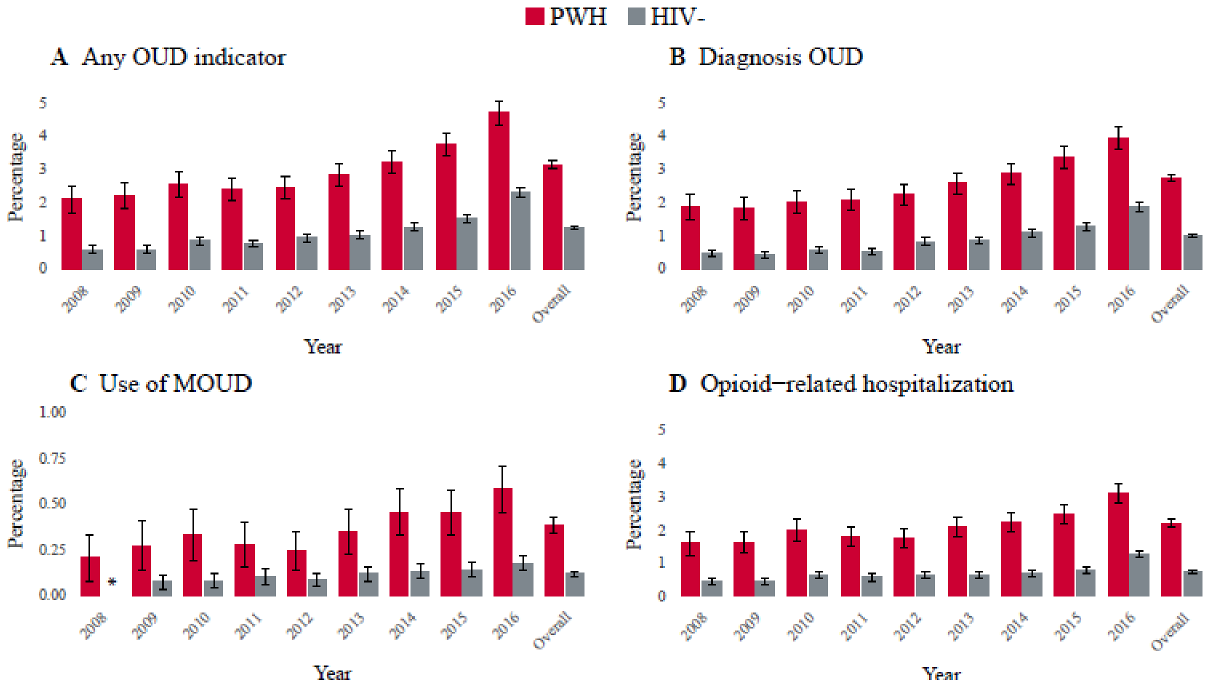

The figure panels are as follows, percentage of beneficiaries (during a calendar year) with: A) any indicator of OUD (composite variable), B) ICD-9-CM/ICD-10-CM diagnosis of OUD, C) use of medication for OUD, D) opioid-related hospitalizations or emergency department visits. Data marked with * indicates censoring due to n<12.

Abbreviations: HIV: human immunodeficiency virus, HIV-: HIV-negative, MOUD: medication for OUD, OUD: opioid use disorder, PWH: people living with HIV.

Supplementary Table 1. Medicare Part D and Chronic Conditions codes and associated study outcomes

Outcome	Database
Primary outcomes	
Receipt of one of more opioid prescriptions	Part D Event file
Indicators of OUD	Master beneficiary file: other chronic conditions summary segment
Secondary outcomes	
High-risk opioid prescriptions:	
Receipt of ≥2 overlapping opioid prescriptions for more than 7 days	Part D Event file
≥1 incident of daily MME ≥90 longer than 7 consecutive days	Part D Event file
≥1 incident of MME ≥120 longer than 7 consecutive days	Part D Event file
≥90 consecutive days of opioid prescription coverage	Part D Event file
Opioid-related sub-indicators:	
Diagnosis and procedure basis for OUD	Master beneficiary file: other chronic conditions summary segment
Use of MOUD	Master beneficiary file: other chronic conditions summary segment
Opioid-related hospitalizations or ED visits	Master beneficiary file: other chronic conditions summary segment

Abbreviations: ED: emergency department, MME: morphine milligram equivalent, MOUD: medication for OUD, OUD: opioid use disorder

Supplementary Table 2. Coding rubric for opioid use disorder outcomes

Condition	Number and Type of Claims	ICD-9/MS DRG/HCPCS Codes	ICD-10/CPT4/HCPCS Codes
Any OUD	Any positive result from the three sub- indicators below.		
Diagnosis OUD	At least 1 inpatient claim OR 2 other non-drug claims of any service type with DX (or ICD-10-PCS procedure) codes in two year lookback period.	DX 304.0, 304.00, 304.01, 304.02, 304.7, 304.70, 304.71, 304.72, 305.5, 305.50, 305.51, 305.52, 760.72, 965.0, 965.00, 965.01, 965.02, 965.09, E85.00, E85.01, E85.02, E93.50, E93.51 (any DX on the claim)	DX F11.10, F11.120, F11.121, F11.122, F11.129, F11.13, F11.14, F11.150, F11.151, F11.159, F11.181, F11.182, F11.188, F11.19, F11.20, F11.220, F11.221, F11.222, F11.229, F11.23, F11.24, F11.250, F11.251, F11.259, F11.281, F11.282, F11.288, F11.29, F11.90, F11.920, F11.921, F11.922, F11.929, F11.93, F11.94, F11.950, F11.951, F11.959, F11.981, F11.982, F11.988, F11.99, T40.0X1A, T40.0X2A, T40.0X3A, T40.0X4A, T40.1X1A, T40.1X2A, T40.1X3A, T40.1X4A, T40.2X1A, T40.2X2A, T40.2X3A, T40.2X4A, T40.3X1A, T40.3X2A, T40.3X3A, T40.3X4A, T40.3X5A, T40.4X1A, T40.4X2A, T40.4X3A, T40.4X4A, T40.411A, T40.412A, T40.413A, T40.414A, T40.415A, T40.421A, T40.422A, T40.423A, T40.424A, T40.425A, T40.491A, T40.492A, T40.493A, T40.494A, T40.495A, T40.601A, T40.602A, T40.603A, T40.604A, T40.691A, T40.692A, T40.693A, T40.694A<br><br>ICD-10 Procedure Codes: HZ81ZZZ, HZ84ZZZ, HZ85ZZZ, HZ86ZZZ, HZ91ZZZ, HZ94ZZZ, HZ95ZZZ, HZ96ZZZ (any position on the claim)
Opioid-related Hospitalization	One inpatient claim OR one emergency department (ED) claim in two year lookback period.	DX 304.00, 304.01, 304.02, 304.70, 304.71, 304.72, 305.50, 305.51, 305.52, 965.00, 965.01, 965.02, 965.09, 970.1, E85.00, E85.01, E85.02, E93.50, E93.51, E93.52, E94.01 (any DX on the claim)	DX F11.10, F11.120, F11.121, F11.122, F11.129, F11.13, F11.14, F11.150, F11.151, F11.159, F11.181, F11.182, F11.188, F11.19, F11.20, F11.220, F11.221, F11.222, F11.229, F11.23, F11.24, F11.250, F11.251, F11.259, F11.281, F11.282, F11.288, F11.29, F11.90, F11.920, F11.921, F11.922, F11.929, F11.93, F11.94, F11.950, F11.951, F11.959, F11.981, F11.982, F11.988, F11.99, T40.0X1A, T40.0X1D, T40.0X1S, T40.0X2A, T40.0X2D, T40.0X2S, T40.0X3A, T40.0X3D, T40.0X3S, T40.0X4A, T40.0X4D, T40.0X4S, T40.0X5A, T40.0X5D, T40.0X5S, T40.1X1A, T40.1X1D, T40.1X1S, T40.1X2A, T40.1X2D, T40.1X2S, T40.1X3A, T40.1X3D, T40.1X3S, T40.1X4A, T40.1X4D, T40.1X4S, T40.2X1A, T40.2X1D, T40.2X1S, T40.2X2A, T40.2X2D, T40.2X2S, T40.2X3A, T40.2X3D, T40.2X3S, T40.2X4A, T40.2X4D, T40.2X4S, T40.2X5A, T40.2X5D, T40.2X5S, T40.3X1A, T40.3X1D, T40.3X1S, T40.3X2A, T40.3X2D, T40.3X2S, T40.3X3A, T40.3X3D, T40.3X3S, T40.3X4A, T40.3X4D, T40.3X4S, T40.3X5A, T40.3X5D, T40.3X5S, T40.4X1A, T40.4X1D, T40.4X1S, T40.4X2A, T40.4X2D, T40.4X2S, T40.4X3A, T40.4X3D, T40.4X3S, T40.4X4A, T40.4X4D, T40.4X4S, T40.4X5A, T40.4X5D, T40.4X5S, T40.411A, T40.411D, T40.411S, T40.412A, T40.412D, T40.412S, T40.413A, T40.413D, T40.413S, T40.414A, T40.414D, T40.414S, T40.415A, T40.415D, T40.415S, T40.421A, T40.421D, T40.421S, T40.422A, T40.422D, T40.422S, T40.423A, T40.423D, T40.423S, T40.424A, T40.424D, T40.424S, T40.425A, T40.425D, T40.425S, T40.491A, T40.491D, T40.491S, T40.492A, T40.492D, T40.492S, T40.493A, T40.493D, T40.493S, T40.494A, T40.494D, T40.494S, T40.495A, T40.495D, T40.495S, T40.601A, T40.601D, T40.601S, T40.602A, T40.602D, T40.602S, T40.603A, T40.603D, T40.603S, T40.604A, T40.604D, T40.604S, T40.605A, T40.605D, T40.605S, T40.691A, T40.691D, T40.691S, T40.692A, T40.692D, T40.692S, T40.693A, T40.693D, T40.693S, T40.694A, T40.694D, T40.694S, T40.695A, T40.695D, T40.695S (any position on the claim)
Use of MOUD	One or more drug claim with an NDC for opioid-MAT OR one or more non-drug	Same as ICD-10	HCPCS codes for MAT:<br><br>G2067, G2068, G2069, G2070, G2071, G2072, G2073, G2078, G2079, H0020, J0571, J0572, J0573, J0574, J0575, J0577, J0578, J0592, J1230, J2315, Q9991, Q9992, S0109

	claim with a HCPCS code in two-year lookback period.<br><br>Naltrexone NDCs are excluded if there is evidence of an alcohol or other drug use disorder. That is, this excludes beneficiaries with NDC for Naltrexone, if the CCW alcohol use disorder indicator = Yes and opioid use disorder DX indicator (from measure Diagnosis OUD) = No or CCW drug use disorder indicator = Yes and opioid use DX disorder = No		NDCs for Buprenorphine:<br><br>00054017613, 00054017713, 00054018813, 00054018913, 00093537856, 00093537956, 00093572056, 00093572156, 00121101830, 00121101930, 00121203630, 00121203830, 00228315303, 00228315403, 00228315473, 00228315503, 00228315567, 00228315573, 00228315603, 00378092393, 00378092493, 00378876516, 00378876593, 00378876616, 00378876693, 00378876716, 00378876793, 00378876816, 00378876893, 00406192303, 00406192403, 00406800503, 00406802003, 00490005100, 00490005130, 00490005160, 00490005190, 00781721606, 00781721664, 00781722706, 00781722764, 00781723806, 00781723864, 00781724906, 00781724964, 00904700906, 00904701006, 00904715404, 00904715504, 12496010001, 12496010002, 12496010005, 12496030001, 12496030002, 12496030005, 12496120201, 12496120203, 12496120401, 12496120403, 12496120801, 12496120803, 12496121201, 12496121203, 12496127802, 12496128302, 12496130602, 12496131002, 16590066605, 16590066630, 16590066705, 16590066730, 16590066790, 16729054910, 16729055010, 23490927003, 23490927006, 23490927009, 35356000407, 35356000430, 35356055530, 35356055630, 42291017430, 42291017530, 42858050103, 42858050203, 42858060103, 42858060203, 43063018407, 43063018430, 43063066706, 43063075306, 43598057901, 43598057930, 43598058001, 43598058030, 43598058101, 43598058130, 43598058201, 43598058230, 47781035503, 47781035511, 47781035603, 47781035611, 47781035703, 47781035711, 47781035803, 47781035811, 49999039507, 49999039515, 49999039530, 49999063830, 49999063930, 50090157100, 50090292400, 50268014411, 50268014415, 50268014511, 50268014515, 50383028793, 50383029493, 50383092493, 50383093093, 50742036401, 50742036430, 50742036501, 50742036530, 50742037201, 50742037204, 50742037301, 50742037304, 50742037401, 50742037404, 50742037501, 50742037504, 50742037601, 50742037604, 51862060830, 52427069203, 52427069211, 52427069403, 52427069411, 52427069803, 52427069811, 52427071203, 52427071211, 52440010014, 52959030430, 52959074930, 53217013830, 53217024630, 54123011430, 54123090730, 54123091430, 54123092930, 54123095730, 54123098630, 54569549600, 54569573900, 54569573901, 54569573902, 54569639900, 54569640800, 54569657800, 54868570700, 54868570701, 54868570702, 54868570703, 54868570704, 54868575000, 55045378403, 55700014730, 55700018430, 55700030230, 55700030330, 55700090130, 58284010014, 58284020801, 58284020891, 58284021601, 58284021691, 58284022401, 58284022491, 58284022801, 58284022891, 58284023201, 58284023291, 58284026401, 58284026491, 58284029601, 58284029691, 59385001201, 59385001230, 59385001401, 59385001430, 59385001601, 59385001630, 60429058611, 60429058630, 60429058633, 60429058711, 60429058730, 60429058733, 60687048111, 60687048121, 60687049211, 60687049221, 60687062611, 60687062665, 60687063711, 60687063765, 62175045232, 62175045832, 62756045983, 62756046083, 62756096983, 62756097083, 63629402801, 63629403401, 63629403402, 63629403403, 63629409201, 63629947501, 63629948201, 63629948301, 63874108403, 63874108503, 63874117303, 65162041503, 65162041603, 66336001630, 68071138003, 68071151003, 68258299103, 68258299903, 68308020230, 68308020830, 70518100700, 70518201400, 70518201401, 70518221700, 70518222600, 70518222602, 70518222603, 70518312900, 70518338900, 71335095001, 71335095002, 71335095003, 71335115401, 71335115402, 71335115403, 71335116301, 71335116302, 71335116303, 71335129601, 71335137801, 71335172501, 71335172502, 71335185801, 71335185802<br><br>NDCs for Naltrexone:

			00056001122, 00056001130, 00056001170, 00056007950, 00056008050, 00185003901, 00185003930, 00406009201, 00406009203, 00406117001, 00406117003, 00555090201, 00555090202, 00904703604, 16729008101, 16729008110, 23155088601, 23155088603, 42291063230, 43063059115, 47335032683, 47335032688, 50090286600, 50090492500, 50090682000, 50436010501, 51224020630, 51224020650, 51285027501, 51285027502, 52152010502, 52152010504, 52152010530, 54868557400, 62135024230, 62135024290, 63459030042, 63629104601, 63629104701, 65694010003, 65694010010, 65757030001, 65757030202, 68084029111, 68084029121, 68094085362, 68115068030, 71300660003, 71300661003, 71300664603, 71300664606, 71300664703, 71300664706, 71300664709, 72162156601, 72162156603, 72162215403

Abbreviations: CCW: chronic conditions warehouse, ED: emergency department, HCPCS: Healthcare Common Procedures Coding System, ICD: International Classifications of Diseases MME: morphine milligram equivalent, MAT: medication assisted treatment, MOUD: medication for OUD, NDC: OUD: opioid use disorder, PCS:

Supplementary Table 3. Characteristics of beneficiaries included in the analysis, by HIV status and year (2008-2021)[a]

	2008		2009		2010		2011		2012		2013		2014	
	PWH	HIV-	PWH	HIV-	PWH	HIV-	PWH	HIV-	PWH	HIV-	PWH	HIV-	PWH	HIV-
**N**	4,849	14,547	5,510	16,530	6,313	18,939	7,169	21,507	8,218	24,654	9,475	28,425	10,505	31,515
**Characteristic**														
**Sex**, n (%)														
Female	1530 (31·6)	4590 (31·6)	1692 (30·7)	5076 (30·7)	1934 (30·6)	5802 (30·6)	2180 (30·4)	6540 (30·4)	2419 (29·4)	7257 (29·4)	2685 (28·3)	8055 (28·3)	3005 (28·6)	9015 (28·6)
Male	3319 (68·4)	9957 (68·4)	3818 (69·3)	11454 (69·3)	4379 (69·4)	13137 (69·4)	4989 (69·6)	14967 (69·6)	5799 (70·6)	17397 (70·6)	6790 (71·7)	20370 (71·7)	7500 (71·4)	22500(71·4)
**Age in years**, mean (SD)	71·2 (5·3)	71·2 (5·3)	71·1 (5·2)	71·1 (5·2)	71·2 (5·3)	71·2 (5·3)	71·3 (5·3)	71·3 (5·3)	71·2 (5·2)	71·2 (5·2)	71·2 (5·1)	71·2 (5·1)	71·2 (5·1)	71·2 (5·1)
**Age in years**, n(%)														
65 – 69	2348 (48·4)	7044 (48·4)	2722 (49·4)	8166 (49·4)	3041 (48·2)	9123 (48·2)	3405 (47·5)	10215 (47·5)	3964 (48·2)	11892 (48·2)	4480 (47·3)	13441 (47·3)	4988 (47·5)	14966 (47·5)
70 – 74	1487 (30·7)	4458 (30·7)	1649 (29·9)	4945 (29·9)	1944 (30·8)	5832 (30·8)	2236 (31·2)	6709 (31·2)	2560 (31·2)	7683 (31·2)	3032 (32·0)	9095 (32·0)	3310 (31·5)	9930 (31·5)
75 – 79	611 (12·6)	1837 (12·6)	704 (12·8)	2114 (12·8)	826 (13·1)	2478 (13·1)	949 (13·2)	2848 (13·2)	1051 (12·8)	3150 (12·8)	1240 (13·1)	3720 (13·1)	1382 (13·2)	4142 (13·1)
80+	403 (8·3)	1208 (8·3)	435 (7·9)	1305 (7·9)	502 (8·0)	1506 (8·0)	579 (8·1)	1735 (8·1)	643 (7·8)	1929 (7·8)	723 (7·6)	2169 (7·6)	825 (7·9)	2477 (7·9)
**Race and Ethnicity**, n(%)[b]														
White	1977 (40·8)	5931 (40·8)	2187 (39·7)	6561 (39·7)	2515 (39·8)	7545 (39·8)	2911 (40·6)	8733 (40·6)	3442 (41·9)	10326 (41·9)	4052 (42·8)	12156 (42·8)	4645 (44·2)	13935 (44·2)
Black	1984 (40·9)	5952 (40·9)	2282 (41·4)	6846 (41·4)	2607 (41·3)	7821 (41·3)	2918 (40·7)	8754 (40·7)	3243 (39·5)	9729 (39·5)	3770 (39·8)	11310 (39·8)	4110 (39·1)	12330 (39·1)
Hispanic	761 (15·7)	2283 (15·7)	912 (16·6)	2736 (16·6)	1035 (16·4)	3105 (16·4)	1170 (16·3)	3510 (16·3)	1305 (15·9)	3915 (15·9)	1378 (14·5)	4134 (14·5)	1399 (13·3)	4197 (13·3)
Asian/Pacific Islander	75 (1·6)	225 (1·6)	80 (1·5)	240 (1·5)	99 (1·6)	297 (1·6)	100 (1·4)	300 (1·4)	115 (1·4)	345 (1·4)	118 (1·3)	354 (1·3)	132 (1·3)	396 (1·3)
Other	52 (1·1)	156 (1·1)	49 (0·9)	147 (0·9)	57 (1·0)	171 (1·0)	70 (1·0)	210 (1·0)	113 (1·4)	339 (1·3)	157 (1·7)	471 (1·7)	219 (2·1)	657 (2·1)
**Dual Eligibility Status**, n(%)[c]	3037 (62·6)	9111(62·6)	3491 (63·4)	10473 (63·4)	4015 (63·6)	12045 (63·6)	4514 (63·0)	13542 (63·0)	4989 (60·7)	14967 (60·7)	5380 (56·8)	16140 (56·8)	5680 (54·1)	17040 (54·1)
**US Census Region**, n(%)[d]														
Northeast	1475 (30·4)	4425 (30·4)	1727 (31·3)	5181 (31·3)	1976 (31·3)	5928 (31·3)	2199 (30·7)	6597 (30·7)	2649 (32·2)	7947 (32·2)	3004 (31·7)	9012 (31·7)	3295 (31·4)	9885 (31·4)
Midwest	493 (10·2)	1479 (10·2)	552 (10·0)	1656 (10·0)	626 (9·9)	1878 (9·9)	723 (10·1)	2169 (10·1)	837 (10·2)	2511 (10·2)	1069 (11·3)	3207 (11·3)	1231 (11·7)	3693 (11·7)
South	2051 (42·3)	6153 (42·3)	2266 (41·1)	6798(41·1)	2587 (41·0)	7761 (41·0)	2988 (41·7)	8964 (41·7)	3339 (40·6)	10017 (40·6)	3804 (40·2)	11412 (40·2)	4189 (39·9)	12567 (39·9)
West	812 (16·8)	2436 (16·8)	948 (17·2)	2844 (17·2)	1107 (17·5)	3321 (17·5)	1234 (17·2)	3702 (17·2)	1367 (16·6)	4101 (16·6)	1577 (16·6)	4731 (16·6)	1768 (16·8)	5304 (16·8)
Other	18 (0·4)	54 (0·4)	17 (0·3)	51 (0·3)	17 (0·3)	51 (0·3)	25 (0·4)	75 (0·4)	26 (0·3)	78 (0·3)	21 (0·2)	63 (0·2)	22 (0·2)	66 (0·2)
**Rural-Urban Community Area,** n(%)														
Metropolitan	4445 (91·8)	12319 (84·8)	5044 (91·6)	13998 (84·8)	5777 (91·6)	16054 (84·9)	6565 (91·7)	18360 (85·5)	7510 (91·5)	20980 (85·2)	8635 (91·2)	24300 (85·6)	9573 (91·2)	26835 (85·2)
Micropolitan	241 (5·0)	1180 (8·1)	277 (5·0)	1302 (7·9)	314 (5·0)	1518 (8·0)	358 (5·0)	1653 (7·7)	397 (4·8)	1953 (7·9)	454 (4·8)	2145 (7·6)	515 (4·9)	2501 (7·9)
Small Town	96 (2·0)	622 (4·3)	111 (2·0)	798 (4·8)	127 (2·0)	850 (4·5)	137 (1·9)	903 (4·2)	181 (2·2)	1054 (4·3)	213 (2·3)	1179 (4·2)	244 (2·3)	1342 (4·3)
Rural	59 (1·2)	404 (2·8)	75 (1·4)	417 (2·5)	88 (1·4)	495 (2·6)	98 (1·4)	558 (2·6)	119 (1·5)	629 (2·6)	165 (1·7)	776 (2·7)	162 (1·5)	802 (2·6)
**ECI,** mean (SD)[e]	3·4 (2·6)	2·9 (2·4)	3·5 (2·7)	3·0 (2·4)	3·6 (2·7)	3·1 (2·5)	3·6 (2·8)	3·1 (2·5)	3·6 (2·8)	3·0 (2·5)	3·5 (2·8)	2·9 (2·5)	3·5 (2·8)	3·0 (2·5)
**ECI,** n(%)														
0	512 (10·6)	2214 (15·2)	521 (9·5)	2228 (13·5)	538 (8·5)	2651 (14·0)	644 (9·0)	3125 (14·5)	776 (9·4)	3673 (14·9)	964 (10·2)	4672 (16·4)	1009 (9·6)	4982 (15·8)
1	805 (16·6)	2499 (17·2)	893 (16·2)	2742 (16·6)	1082 (17·1)	3031 (16·0)	1197 (16·7)	3542 (16·5)	1335 (16·2)	4004 (16·2)	1577 (16·6)	4779 (16·8)	1745 (16·6)	5189 (16·5)
2	832 (17·2)	2767 (19·0)	938 (17·0)	3185 (19·3)	1041 (16·5)	3499 (18·5)	1215 (17·0)	3904 (18·2)	1389 (16·9)	4535 (18·4)	1638 (17·3)	5213 (18·3)	1824 (17·4)	5709 (18·1)
3	679 (14·0)	2282 (15·7)	824 (15·0)	2642 (16·0)	960 (15·1)	2945 (15·6)	1051 (14·7)	3293 (15·3)	1224 (14·9)	3795 (15·4)	1379 (14·6)	4342 (15·3)	1523 (14·5)	4930 (15·6)
4-5	1039 (21·4)	2763 (19·0)	1174 (21·3)	3230 (195)	1342 (21·3)	3793 (20·0)	1446 (20·2)	4175 (19·4)	1677 (20·4)	4788 (19·4)	1908 (20·1)	5201 (18·3)	2159 (20·6)	5969 (18·9)

6+	982 (20·3)	2022 (13·9)	1174 (21·1	2503 (15.1)	1350 (21·4)	3020 (16·0)	1616 (22·5)	3468 (16·1)	1817 (22·1)	3859 (15·7)	2009 (21·2)	4218 (14·8)	2245 (21·4)	4736 (15·0
**Death during year,** n(%)	485 (10.0)	744 (5.2)	522 (9.5)	920 (5.6)	538 (8.5)	1059 (5.6)	592 (8.3)	1044 (4.9)	610 (7.4)	1208 (4.9)	685 (7.2)	1340 (4.7)	781 (7.4)	1563 (5.0)

	2015		2016		2017		2018		2019		2020		2021	
	PWH	HIV-	PWH	HIV-	PWH	HIV-	PWH	HIV-	PWH	HIV-	PWH	HIV-	PWH	HIV-
**N**	11,518	34,554	13,080	39,240	14,501	43,503	15,943	47,829	17,407	52,221	18,955	56,865	19,986	59,958
**Characteristic**														
**Sex**, n(%)														
Female	3211 (27·9)	9633 (27·9)	3669 (28·1)	11007 (28·1)	3932 (27·1)	11796 (27·1)	4156 (26·1)	12468 (26·1)	4466 (25·7)	13398 (25·7)	4751 (25·1)	14253 (25·1)	4920 (24·6)	14760 (24·6)
Male	8307 (72·1)	24921 (72·1)	9411 (71·9)	28233 (71·9)	10569 (72·9)	31707 (72·9)	11787 (73·9)	35361 (73·9)	12941 (74·3)	38823 (74·3)	14204 (74·9)	42612 (74·9)	15066 (75·4)	45198 (75·4)
**Age in years**, mean (SD)	71·3 (5·2)	71·3 (5·2)	71·3 (5·4)	71·3 (5·4)	71·3 (5·3)	71·3 (5·3)	71·2 (5·2)	71·2 (5·2)	71·3 (5·2)	71·3 (5·2)	71·6 (5·1)	71·6 (5·1)	71·9 (5·2)	71·9 (5·2)
**Age in years**, n(%)														
65 – 69	5495 (47·7)	16483 (47·7)	6332 (48·4)	19001 (48·4)	6986 (48·2)	20956 (48·2)	7687 (48·2)	23063 (48·2)	8258 (47·4)	24776 (47·4)	8298 (43·8)	24899 (43·8)	8058 (40·3)	24179 (40·3)
70 – 74	3535 (30·7)	10607 (30·7)	3906 (29·9)	11713 (29·9)	4316 (29·8)	12952 (29·8)	4793 (30·1)	14381 (30·1)	5384 (30·9)	16151 (30·9)	6380 (33·7)	19135 (33·7)	7108 (35·6)	21317 (35·6)
75 – 79	1566 (13·6)	4698 (13·6)	1750 (13·4)	5251 (13·4)	1978 (13·6)	5932 (13·6)	2168 (13·6)	6500 (13·6)	2327 (13·4)	6980 (13·4)	2657 (14·0)	7971 (14·0)	2996 (15·0)	8987 (15·0)
80+	922 (8·0)	2766 (8·0)	1092 (8·4)	3275 (8·4)	1221 (8·4)	3663 (8·4)	1295 (8·1)	3885 (8·1)	1438 (8·3)	4313 (8·3)	1620 (8·6)	4860 (8·6)	1824 (9·1)	5473 (9·1)
**Race and Ethnicity**, n(%)[b]														
White	5231 (45·4)	15693 (45·4)	6085 (46·5)	18255 (46·5)	6857 (47·3)	20571 (47·3)	7695 (48·3)	23085 (48·3)	8521 (49·0)	25563 (49·0)	9636 (50·8)	28908 (50·8)	10690 (53·5)	32070 (53·5)
Black	4359 (37·9)	13077 (37·9)	4843 (37·0)	14529 (37·0)	5294 (36·5)	15882 (36·5)	5650 (35·4)	16950 (35·4)	6042 (34·7)	18126 (34·7)	6275 (33·1)	18825 (33·1)	6166 (30·9)	18498 (30·9)
Hispanic	1488 (12·9)	4464 (12·9)	1639 (12·5)	4917 (12·5)	1733 (12·0)	5199 (12·0)	1887 (11·8)	5661 (11·8)	2033 (11·7)	6099 (11·7)	2145 (11·3)	6435 (11·3)	2147 (10·7)	6441 (10·7)
Asian/Pacific Islander	148 (1·3)	444 (1·3)	161 (1·2)	483 (1·2)	180 (1·2)	540 (1·2)	204 (1·3)	612 (1·3)	258 (1·5)	774 (1·5)	269 (1·4)	807 (1·4)	302 (1·5)	906 (1·5)
Other	292 (2·5)	876 (2·4)	352 (2·7)	1056 (2·7)	437 (3·0)	1311 (3·0)	507 (2·2)	1521 (2·2)	553 (3·2)	1659 (3·2)	630 (3·3)	1890 (3.3)	681 (3·4)	2043 (3·4)
**Dual Eligibility Status**, n(%)[c]	6032 (52·4)	18096 (52·4)	6718 (51·4)	20154 (51·4)	7366 (50·8)	22098 (50·8)	7873 (49·4)	23619 (49·4)	8386 (48·2)	25158 (48·2)	8872 (46·8)	26616 (46·8)	8981 (44·9)	26943 (44·9)
**US Census Region**, n(%)[d]														
Northeast	10794 (31·2)	3598 (31·2)	3995 (30·5)	11985 (30·5)	4316 (29·8)	12948 (29·8)	4679 (29·4)	14037 (29·4)	4966 (28·5)	14898 (28·5)	5372 (28·3)	16116 (28·3)	5551 (27·8)	16653 (27·8)
Midwest	3708 (10·7)	1236 (10·7)	1402 (10·7)	4206 (10·7)	1627 (11·2)	4881 (11·2)	1781 (11·2)	5343 (11·2)	1932 (11·1)	5796 (11·1)	2130 (11·2)	6390 (11·2)	2231 (11·2)	6693 (11·2)
South	13912 (40·0)	4604 (40·0)	5202 (39·8)	15606 (39·8)	5757 (39·7)	17271 (39·7)	6284 (39·4)	18852 (39·4)	6959 (40·0)	20877 (40·0)	7464 (39·4)	22392 (39·4)	7762 (38·8)	23286 (38·8)
West	6189 (17·9)	2063 (17·9)	2457 (18·8)	7371 (18·8)	2772 (19·1)	8316 (19·1)	3167 (19·9)	9501 (19·9)	3517 (20·2)	10551 (20·2)	3948 (20·8)	11844 (20·8)	4404 (22·0)	13212 (22·0)
Other	51 (0·2)	17 (0·2)	24 (0·2)	72 (0·2)	29 (0·2)	87 (0·2)	32 (0·2)	96 (0·2)	33 (0·2)	99 (0·2)	41 (0·2)	123 (0·2)	38 (0·2)	114 (0·2)
**Rural-Urban Community Area**, n(%)														
Metropolitan	10503 (91·3)	29258 (84·8)	11863 (90·8)	33237 (84·8)	13140 (90·7)	36683 (84·4)	14461 (90·8)	40467 (84·7)	15753 (90·6)	44074 (84·5)	17114 (90·4)	47975 (84·5)	18050 (90·4)	50272 (83·9)
Micropolitan	567 (4·9)	2821 (8·2)	677 (5·2)	3173 (8·1)	780 (5·4)	3656 (8·4)	845 (5·3)	3956 (8·3)	956 (5·5)	4370 (8·4)	1047 (5·5)	4744 (8·4)	1104 (5·5)	5155 (8·6)
Small Town	267 (2·3)	1483 (4·3)	312 (2·4)	1735 (4·4)	339 (2·3)	1889 (4·4)	379 (2·4)	2030 (4·3)	406 (2·3)	2252 (4·3)	444 (2·4)	2462 (4·3)	464 (2·3)	2684 (4·5)
Rural	172 (1·5)	960 (2·8)	215 (1·7)	1055 (2·7)	229 (1·6)	1233 (2·8)	242 (1·5)	1326 (2·8)	275 (1·6)	1471 (2·8)	327 (1·7)	1613 (2·8)	350 (1·8)	1792 (3·0)
**ECI,** mean (SD)[e]	4.0 (3·1)	3·3 (2·9)	4·6 (3·6)	3·9 (3·3)	4·7 (3·6)	4·0 (3·3)	4·8 (3·6)	4·0 (3·4)	4·8 (3·6)	4·1 (3·4)	4·6 (3·6)	3·9 (3·3)	4·6 (3·5)	4·0 (3·3)
**ECI,** n(%)														
0	952 (8·3)	5407 (15·7)	932 (7·1)	5459 (13·9)	960 (6·6)	5768 (13·3)	1040 (6·5)	6430 (13·4)	1079 (6·2)	6724 (12·9)	1386 (7·3)	8118 (14·3)	1311 (6·6)	7693 (12·8)
1	1646 (14·3)	5087 (14·7)	1593 (12·1)	4902 (12·5)	1670 (11·5)	5339 (12·3)	1872 (11·7)	5862 (12·3)	1911 (11·0)	6233 (11·9)	2406 (12·7)	7136 (12·6)	2455 (12·3)	7521 (12·5)

2	1878 (16·3)	5657 (16·4)	1825 (14·0)	5559 (14·2)	1992 (13·7)	6183 (14·2)	2107 (13·2)	5461 (13·7)	2382 (13·7)	7311 (14·0)	2643 (13·9)	7964 (14·0)	2833 (14·2)	8621 (14·4)
3	1586 (13·8)	4885 (14·1)	1715 (13·1)	5277 (13·5)	1896 (13·1)	5749 (13·2)	2041 (12·8)	6198 (13·0)	2247 (12·9)	6801 (13·0)	2436 (12·9)	7425 (13·1)	2654 (13·3)	8074 (13·5)
4-5	2338 (20·3)	6677 (19·3)	2611 (20·0)	7796 (19·9)	2955 (20·4)	8762 (20·1)	3321 (20·8)	9749 (20·4)	3644 (20·9)	10601 (20·3)	3872 (20·4)	11022 (19·4)	4176 (20·9)	12063 (20·1)
6+	3118 (27·1)	6841 (19·8)	4404 (33·7)	10247 (26·1)	5028 (35·7)	11702 (26·9)	5562 (34·9)	13029 (27·3)	6143 (35·3)	14551 (27·9)	6212 (32·8)	15200 (26·7)	6557 (32·8)	15986 (26·7)
**Death during year,** n(%)	781 (6.8)	1595 (4.6)	918 (7.0)	1834 (4.7)	976 (6.7)	1996 (4.6)	1001 (6.3)	2051 (4.3)	1029 (5·9)	2307 (4·4)	1306 (6·9)	3317 (5·8)	1285 (6·4)	2990 (5·0)

[a] Beneficiaries were matched on sex, age, race and ethnicity, state, and dual eligibility status.

[b] Race and ethnicity were abstracted from Centers for Medicare and Medicaid Services beneficiary data. The category ‘Other’ represents North American Native, Unknown, and other races and ethnicities.

[c] ‘Dual Eligibility Status’ means an individual was eligible for both Medicaid and Medicare.

[d] The category ‘other’ of US Census region refers to territories outside of the 50 states.

[e] The Elixhauser Comorbidity Index was used to code for comorbidities, with each comorbid condition being identified using the International Classification of Diseases 9th and 10th revision diagnosis codes.

Abbreviations: ECI: Elixhauser Comorbidity Index, HIV: human immunodeficiency virus, HIV-: HIV-negative, PWH: people living with HIV.

Supplementary Table 4. Average annual prevalence and overall odds ratios (OR) and 95% confidence interval (95% CI) of opioid prescription measures and indicators of OUD in Medicare beneficiaries with HIV compared to HIV-negative beneficiaries for all years combined (2008-2021)[a]

**Outcomes**	**% (95% CI)**		**OR (95% CI)**	**OR (95% CI)**
	PWH	HIV-	PWH vs HIV-	one-year increase
1+ opioid prescription	35·11 (34·87, 35·34)	28·26 (28·13, 28·38)	1·38 (1·36, 1·39)	0·95 (0·95, 0·95)
≥1 incident of overlapping opioid prescriptions longer than 7 consecutive days	5·30 (5·19, 5·41)	3·47 (3·42, 3·52)	1·56 (1·52, 1·60)	0·92 (0·92, 0·92)
≥1 incident of total daily MMEs ≥ 90 for longer than 7 consecutive days	4·03 (3·93, 4·12)	2·24 (2·20, 2·28)	1·84 (1·78, 1·89)	0·91 (0·91, 0·91)
≥1 incident of total daily MMEs ≥120 for longer than 7 consecutive days	3·06 (2·98, 3·14)	1·55 (1·51, 1·58)	2·01 (1·94, 2·09)	0·92 (0·92, 0·92)
≥ 90 consecutive days of opioid coverage	6·08 (5·97, 6·20)	3·93 (3·88, 3·99)	1·58 (1·54, 1·62)	0·98 (0·98, 0·98)
Any OUD indicator	3·14 (3·02, 3·27)	1·23 (1·19, 1·28)	2·61 (2·47, 2·76)	1·16 (1·15, 1·18)
Any OUD indicator – except MOUD	2·76 (2·64, 2·87)	1·12 (1·07, 1·16)	2·52 (2·37, 2·67)	1·17 (1·15, 1·18)
Diagnosed OUD	2·74 (2·62, 2·85)	1·00 (0·96, 1·04)	2·78 (2·62, 2·95)	1·16 (1·16, 1·19)
MOUD	0·38 (0·34, 0·43)	0·12 (0·10, 0·13)	3·02 (2·82, 3·23)	1·11 (1·09, 1·13)
Opioid related hospitalization or emergency department visit	2·21 (2·11, 2·32)	0·75 (0·71, 0·78)	3·35 (2·84, 3·96)	1·14 (1·10, 1·18)

[a]Overall odds ratios were calculated using generalized estimating equations, adjusting for calendar year, and accounting for repeated beneficiary IDs and matching IDs.

Abbreviations: HIV: human immunodeficiency virus, MME: morphine milligram equivalent, PWH: people living with HIV, MOUD: medication for OUD, OUD: opioid use disorder.

Supplementary Table 5. Percentage of Medicare beneficiaries with HIV and matched HIV-negative beneficiaries receiving one or more opioid prescription, by calendar year

		1+ opioid prescription		≥1 incident of overlapping opioid prescriptions longer than 7 consecutive days		≥1 incident of total daily MMEs ≥ 90 for longer than 7 consecutive days		≥1 incident of total daily MMEs ≥120 for longer than 7 consecutive days		≥ 90 consecutive days of opioid coverage	
		**PWH**	**HIV-**	**PWH**	**HIV-**	**PWH**	**HIV-**	**PWH**	**HIV-**	**PWH**	**HIV-**
**Year**											
2008	n	1984	4808	334	728	299	646	199	400	265	539
PWH: 4849, HIV-: 14547	% (95% CI)	40·92 (39·53, 42·30)	33·05 (32·29, 33·82)	6·89 (6·18, 7·60)	5·00 (4·65, 5·36)	6·17 (5·49, 6·84)	4·44 (4·11, 4·78)	4·10 (3·55, 4·66)	2·75 (2·48, 3·02)	5·47 (4·83, 6·11)	3·71 (3·40, 4·01)
2009	n	2345	5765	431	871	391	805	261	476	360	683
PWH: 5510 HIV-: 16530	% (95% CI)	42·56 (41·25, 43·86)	34·88 (34·15, 35·60)	7·82 (7·11, 8·53)	5·27 (4·93, 5·61)	7·10 (6·42, 7·77)	4·87 (4·54, 5·20)	4·74 (4·18, 5·30)	2·88 (2·62, 3·13)	6·53 (5·88, 7·19)	4·13 (3·83, 4·44)
2010	n	2713	6743	503	1025	427	803	312	508	415	818
PWH: 6313 HIV-: 18939	% (95% CI)	42·97 (41·75, 44·20)	35·60 (34·92, 36·29)	7·97 (7·30, 8·64)	5·41 (5·09, 5·73)	6·76 (6·14, 7·38)	4·24 (3·95, 4·53)	4·94 (4·41, 5·48)	2·68 (2·45 2·91)	6·57 (5·96, 7·19)	4·32 (4·03, 4·61)
2011	n	2963	7336	530	1061	361	573	283	417	484	870
PWH: 7169 HIV-: 21507	% (95% CI)	41·33 (40·19, 42·47)	34·11 (33·48, 34·74)	7·39 (6·79, 8·00)	4·93 (4·64, 5·22)	5·04 (4·53, 5·54)	2·66 (2·45, 2·88)	3·95 (3·50, 4·40)	1·94 (1·75, 2·12)	6·75 (6·17, 7·33)	4·05 (3·78, 4·31)
2012	n	3385	8219	632	1216	416	681	318	494	564	1114
PWH: 8218 HIV-: 24654	% (95% CI)	41·19 (40·13, 42·25)	33·34 (32·75, 33·93)	7·69 (7·11, 8·27)	4·93 (4·66, 5·20)	5·06 (4·59, 5·54)	2·76 (2·56, 2·97)	3·87 (3·45, 4·29)	2·00 (1·83, 2·18)	6·86 (6·32, 7·41)	4·52 (4·26, 4·78)
2013	n	3532	8740	661	1302	420	733	327	549	634	1167
PWH: 9475 HIV-: 28425	% (95% CI)	37·28 (36·30, 38·25)	30·75 (30·21, 31·28)	6·98 (6·46, 7·49)	4·58 (4·34, 4·82)	4·43 (4·02, 4·85)	2·58 (2·39, 2·76)	3·45 (3·08, 3·82)	1·93 (1·77, 2·09)	6·69 (6·19, 7·19)	4·11 (3·87, 4·34)
2014	n	4024	9811	683	1438	503	855	383	624	713	1444
PWH: 10505 HIV-: 31515	% (95% CI)	38·31 (37·38, 39·24)	31·13 (30·62, 31·64)	6·50 (6·03, 6·97)	4·56 (4·33, 4·79)	4·79 (4·38, 5·20)	2·71 (2·53, 2·89)	3·65 (3·29, 4·00)	1·98 (1·83, 2·13)	6·79 (6·31, 7·27)	4·58 (4·35, 4·81)
2015	n	4350	10661	704	1432	522	886	386	603	732	1446
PWH: 11518 HIV-: 34554	% (95% CI)	37·77 (36·88, 38·65)	30·85 (30·37, 31·34)	6·11 (5·67, 6·55)	4·14 (3·93, 4·35)	4·53 (4·15, 4·91)	2·56 (2·40, 2·73)	3·35 (3·02, 3·68)	1·75 (1·61, 1·88)	6·36 (5·91, 6·80)	4·18 (3·97, 4·40)
2016	n	4725	11796	774	1585	580	978	438	705	829	1653

PWH: 13080 HIV-:39240	% (95% CI)	36·12 (35·30, 36·95)	30·06 (29·61, 30·51)	5·92 (5·51, 6·32)	4·04 (3·84, 4·23)	4·43 (4·08, 4·79)	2·49 (2·34, 2·65)	3·35 (3·04, 3·66)	1·80 (1·67, 1·93)	6·34 (5·92, 6·76)	4·21 (4·01, 4·41)
2017	n	5232	12584	786	1552	572	928	457	625	931	1845
PWH: 14501 HIV-: 43503	% (95% CI)	36·08 (35·30, 36·95)	28·93 (28·50, 29·35)	5·42 (5·05, 5·79)	3·57 (3·39, 3·74)	3·94 (3·63, 4·26)	2·13 (2·00, 2·27)	3·15 (2·87, 3·44)	1·44 (1·32, 1·55)	6·42 (6·02, 6·82)	4·24 (4·04, 4·43)
2018	n	5365	12896	692	1335	551	870	423	598	900	1838
PWH: 15943 HIV-: 47829	% (95% CI)	33·65 (32·92, 34·38)	26·96 (26·57, 27·36)	4·34 (4·02, 4·66)	2·79 (2·64, 2·94)	3·46 (3·17, 3.74)	1·82 (1·70, 1·94)	2·65 (2·40, 2·90)	1·25 (1·15, 1·35)	5·65 (5·29, 6·00)	3·84 (3·67, 4·02)
2019	n	5514	12997	667	1196	526	739	410	536	1008	1877
PWH: 17407 HIV-: 52221	% (95% CI)	31·68 (30·99, 32·37)	24·89 (24·52, 25·26)	3·83 (3·55, 4·12)	2·29 (2·16, 2·42)	3·02 (2·77, 3·28)	1·42 (1·31, 1·52)	2·36 (2·13, 2·58)	1·03 (0·94, 1·11)	5·79 (5·44, 6·14)	3·59 (3·43, 3·75)
2020	n	5444	12964	658	1215	526	794	419	563	1062	2050
PWH: 18955 HIV-: 56865	% (95% CI)	28·72 (28·08, 29·40)	22·80 (22·45, 23·14)	3·47 (3·21, 3·73)	2·14 (2·02, 2·26)	2·78 (2·54, 3·01)	1·40 (1·30, 1·49)	2·21 (2·00, 2·42)	0·99 (0·91, 1·07)	5·60 (5·28, 5·93)	3·61 (3·45, 3·76)
2021	n	5797	13227	603	1075	485	684	385	481	1044	1936
PWH: 19986 HIV-: 59958	% (95% CI)	29·01 (28·38, 29·63)	22·06 (21·73, 22·39)	3·02 (2·78, 3·25)	1·79 (1·69, 1·90)	2·43 (2·21, 2·64)	1·14 (1·06, 1·23)	1·93 (1·74, 2·12)	0·80 (0·73, 0·87)	5·22 (4·92, 5·53)	3·23 (3·09, 3·37)

Abbreviations: CI: confidence interval, HIV: human immunodeficiency virus, HIV-: HIV-negative, MMES: morphine milligram equivalent, PWH: people living with HIV.

Supplementary Table 6. Percentage of Medicare beneficiaries with HIV and matched HIV-negative beneficiaries with opioid use disorder outcomes, by calendar year

		Any OUD indicator		Any OUD indicator – except MOUD		Diagnosed OUD		MOUD		Opioid related hospitalization or emergency department visit	
		**PWH**	**HIV-**	**PWH**	**HIV-**	**PWH**	**HIV-**	**PWH**	**HIV-**	**PWH**	**HIV-**
**Year**											
2008	n	101	82	91	78	90	67	10	<12	77	66
PWH: 4849 HIV-: 14547	% (95% CI)	2·08 (1·68, 2·49)	0·56 (0·44, 0·69)	1·88 (1·49, 2·26)	0·54 (0·42, 0·65)	1·86 (1·48, 2·24)	0·46 (0·35, 0·57)	0·21 (0·08, 0·33)	-	1·59 (1·24, 1·94)	0·45 (0·34, 0·56)
1 Indicator	n	26	28								
	% (95% CI)	25·7 (17·07, 34.42)	34·15 (23·66, 44·63)								
2+ Indicators	n	75	54								
	% (95% CI)	74·26 (65·58, 82·93)	65·85 (55·37, 76·34)								
2009	n	121	96	106	84	100	68	15	12	90	76
PWH: 5510 HIV-:16530	% (95% CI)	2·20 (1·81, 2·58)	0·58 (0·46, 0·70)	1·92 (1·56, 2·29)	0·50 (0·40, 0·62)	1·81 (1·46, 2·17)	0·41 (0·31, 0·51)	0·27 (0·13, 0·41)	0·07 (0·03, 0·11)	1·63 (1·30, 1·97)	0·46 (0·36, 0·56)
1 Indicator	n	41	41								
	% (95% CI)	33·88 (25·33, 42·44)	42·71 (32.63, 52·78)								
2+ Indicators	n	80	55								
	% (95% CI)	66·12 (57·56, 74·67)	57·29 (47.22, 67·37)								
2010	n	161	157	140	142	126	106	21	15	125	122
PWH: 6313 HIV-:18939	% (95% CI)	2·55 (2·16, 2·94)	0·83 (0·70, 0·96)	2·22 (1·85, 2·58)	0·75 (0·63, 0·87)	2·00 (1·65, 2·34)	0·56 (0·45, 0·67)	0·33 (0·19, 0·47)	0·08 (0·04, 0·12)	1·98 (1·64, 2·32)	0·64 (0·53, 0·76)
1 Indicator	n	58	76								
	% (95% CI)	36·02 (28·53, 43·50)	48·41 (40·50, 56·31)								
2+ Indicators	n	103	81								
	% (95% CI)	63·98 (56·48, 71·47)	51·59 (43·69, 59·50)								
2011	n	172	161	152	139	149	114	20	22	129	124

PWH: 7169 HIV-:21507	% (95% CI)	2·40 (2·04, 2·75)	0·75 (0·63, 0·86)	2·12 (1·79, 2.45)	0·65 (0·54, 0·75)	2·08 (1·75, 2·41)	0·53 (0·43, 0·63)	0·28 (0·16, 0·40)	0·10 (0·06, 0·15)	1·80 (1·49, 2·11)	0·58 (0·48, 0·68)
1 Indicator	n	55	73								
	% (95% CI)	31·98 (24·94, 39·02)	45·34 (37·57, 53·11)								
2+ Indicators	n	117	88								
	% (95% CI)	68·02 (60·98, 75·06)	54·66 (48·89, 62·43)								
2012	n	200	229	180	208	184	201	20	21	146	155
PWH: 8218 HIV-:24654	% (95% CI)	2·43 (2·10, 2·77)	0·93 (0·81, 1·05)	2·19 (1·87, 2·51)	0·84 (0·73, 0·96)	2·24 (1·92, 2·56)	0·82 (0·70, 0·93)	0·24 (0·14, 0·35)	0·09 (0·05, 0·12)	1·78 (1·49, 2·06)	0·63 (0·53, 0·73)
1 Indicator	n	56	85								
	% (95% CI)	28·00 (21·72, 34·28)	37·12 (30·81, 43·42)								
2+ Indicators	n	144	144								
	% (95% CI)	72·00 (65·72, 78·28)	62·88 (56·58, 69·19)								
2013	n	269	288	236	255	243	243	33	33	200	180
PWH: 9475 HIV-:28425	% (95% CI)	2·84 (2·50, 3·17)	1·01 (0·90, 1·13)	2·49 (2·18, 2·80)	0·90 (0·79, 1·01)	2·56 (2·25, 2·88)	0·85 (0·75, 0·96)	0·35 (0·23, 0·47)	0·12 (0·08, 0·16)	2·11 (1·82, 2·40)	0·63 (0·54, 0·73)
1 Indicator	n	78	132								
	% (95% CI)	29·00 (23·54, 34·45)	45·83 (40·04, 51.62)								
2+ Indicators	n	191	156								
	% (95% CI)	71·00 (65·55, 76·46)	54·17 (48·38, 59·96)								
2014	n	339	390	291	349	301	338	48	41	236	215
PWH: 10505 HIV-:31515	% (95% CI)	3·23 (2·89, 3·57)	1·24 (1·12, 1·36)	2·77 (2·46, 3·08)	1·11 (0·99, 1·22)	2·87 (2·55, 3·18)	1·07 (0·96, 1·19)	0·46 (0·33, 0·59)	0·13 (0·09, 0·17)	2·25 (1·96, 2·53)	0·68 (0·59, 0·77)
1 Indicator	n	113	192								
	% (95% CI)	33·33 (28·29, 38·38)	49·23 (44·25, 54·21)								
2+ Indicators	n	226	198								
	% (95% CI)	66·67 (61·62, 71·71)	50·77 (45·78, 55·75)								
2015	n	432	520	380	472	388	442	52	48	286	274

PWH:11518 HIV-:34554	% (95% CI)	3·7 (3·40, 4·10)	1·50 (1·38, 1·63)	3·30 (2·97, 3·63)	1·37 (1·24, 1·49)	3·37 (3·04, 3·70)	1·28 (1·16, 1·40)	0·45 (0·33, 0·57)	0·14 (0·10, 0·18)	2·48 (2·20, 2·77)	0·79 (0·70, 0·89)
1 Indicator	n	161	294								
	% (95% CI)	37·27 (32·69, 41·85)	56·54 (52·26, 60·81)								
2+ Indicators	n	271	226								
	% (95% CI)	62·73 (58·15, 67·31)	43·46 (39·19, 47·74)								
2016	n	613	908	537	839	517	729	76	69	407	502
PWH: 13080 HIV-:39240	% (95% CI)	4·69 (4·32, 5·05)	2.31 (2·17, 2·46)	4·11 (3·77, 4·45)	2·14 (2·00, 2·28)	3·95 (3·62, 4·29)	1·86 (1·72, 1·99)	0·58 (0·45, 0·71)	0·18 (0·13, 0·22)	3·11 (2·81, 3·41)	1·28 (1·17, 1·39)
1 Indicator	n	262	530								
	% (95% CI)	42·74 (38·81, 46·67)	58·37 (55·16, 61·58)								
2+ Indicators	n	351	378								
	% (95% CI)	57·26 (53·33, 61·19)	41·63 (38·42, 44·84)								

Abbreviations: HIV: human immunodeficiency virus, HIV-: HIV-negative, MMES: morphine milligram equivalent, PWH: people living with HIV.

Supplementary Table 7. Odds ratios of opioid prescription measures in Medicare beneficiaries with HIV compared to matched HIV-negative beneficiaries, unadjusted and adjusted for anxiety, fibromyalgia/chronic pain/fatigue, depression, or ECI, by calendar year

**Year**	2008	2009	2010	2011	2012	2013	2014	2015	2016	2017	2018	2019	2020	2021
**Outcome**	Odds ratio (95% CI)													
**≥1 opioid prescriptions in the last year**														
Adjusted for:														
Unadjusted	1·43 (1·33, 1·53)	1·40 (1·32, 1·50)	1·37 (1·30, 1·46)	1·38 (1·30, 1·46)	1·41 (1·34, 1·49)	1·35 (1·28, 1·42)	1·39 (1·32, 1·45)	1·37 (1·31, 1·43)	1·33 (1·27, 1·39)	1·40 (1·35, 1·46)	1·39 (1·33, 1·44)	1·41 (1·36, 1·47)	1·38 (1·33, 1·43)	1·46 (1·41, 1·51)
Death in Cross-Sectional Year	1·42 (1·33, 1·52)	1·39 (1·30, 1·48)	1·37 (1·29, 1·45)	1·37 (1·30, 1·45)	1·40 (1·33, 1·48)	1·34 (1·28, 1·41)	1·38 (1·32, 1·45)	1·36 (1·30, 1·43)	1·32 (1·26, 1·38)	1·39 (1·34, 1·45)	1·38 (1·32, 1·43)	1·41 (1·36, 1·46)	1·38 (1·32, 1·43)	1·45 (1·40, 1·51)
Anxiety	1·40 (1·31, 1·51)	1·38 (1·30, 1·47)	1·36 (1·28, 1·44)	1·36 (1·28, 1·43)	1·38 (1·31, 1·46)	1·31 (1·24, 1·37)	1·35 (1·29, 1·41)	1·32 (1·26, 1·38)	1·28 (1·22, 1·33)	-	-	-	-	-
Fibromyalgia/ Chronic Pain/Fatigue	1·41 (1·31, 1·51)	1·39 (1·31, 1·49)	1·33 (1·25, 1·41)	1·34 (1·27, 1·42)	1·38 (1·31, 1·45)	1·30 (1·23, 1·37)	1·33 (1·27, 1·40)	1·29 (1·23, 1·35)	1·25 (1·20, 1·31)	-	-	-	-	-
Depression	1·36 (1·27, 1·46)	1·34 (1·26, 1·43)	1·30 (1·23, 1·38)	1·30 (1·23, 1·38)	1·33 (1·26, 1·40)	1·27 (1·21, 1·34)	1·30 (1·24, 1·36)	1·27 (1·21, 1·33)	1·24 (1·18, 1·29)	-	-	-	-	-
ECI	1·27 (1·18, 1·37)	1·27 (1·19, 1·36)	1·25 (1·18, 1·33)	1·25 (1·17, 1·32)	1·28 (1·21, 1·35)	1·20 (1·14, 1·27)	1·24 (1·18, 1·30)	1·21 (1·15, 1·27)	1·18 (1·13, 1·23)	1·25 (1·19, 1·30)	1·23 (1·18, 1·28)	1·26 (1·21, 1·32)	1·26 (1·21, 1·31)	1·33 (1·28, 1·38)
Single ECI[a], locality, and age	1·30 (1·21, 1·41)	1·30 (1·21, 1·39)	1·27 (1·19, 1·36)	1·23 (1·16, 1·31)	1·28 (1·20, 1·35)	1·19 (1·13, 1·26)	1·23 (1·16, 1·29)	1·20 (1·14, 1·27)	1·17 (1·12, 1·23)	1·23 (1·17, 1·29)	1·22 (1·17, 1·28)	1·25 (1·20, 1·30)	1·27 (1·22, 1·33)	1·30 (1·25, 1·35)
**≥1 incidents of overlapping opioid prescriptions longer than 7 days**														
Adjusted for:														
Unadjusted	1·42 (1·24, 1·62)	1·55 (1·37, 1·76)	1·53 (1·37, 1·71)	1·56 (1·40, 1·75)	1·63 (1·47, 1·80)	1·58 (1·43, 1·74)	1·47 (1·34, 1·61)	1·52 (1·38, 1·66)	1·51 (1·38, 1·65)	1·57 (1·43, 1·71)	1·59 (1·45, 1·75)	1·71 (1·55, 1·89)	1·66 (1·51, 1·83)	1·71 (1·55, 1·89)
Death in Cross-Sectional Year	1·39 (1·21, 1·59)	1·54 (1·36, 1·74)	1·51 (1·34, 1·69)	1·55 (1·39, 1·73)	1·61 (1·46, 1·79)	1·56 (1·41, 1·72)	1·46 (1·33, 1·60)	1·50 (1·36, 1·65)	1·49 (1·36, 1·63)	1·55 (1·42, 1·70)	1·58 (1·43, 1·73)	1·70 (1·54, 1·87)	1·66 (1·50, 1·83)	1·71 (1·54, 1·89)
Anxiety	1·37 (1·19, 1·57)	1·52 (1·34, 1·72)	1·49 (1·33, 1·67)	1·52 (1·36, 1·70)	1·57 (1·42, 1·74)	1·51 (1·36, 1·67)	1·42 (1·29, 1·56)	1·43 (1·30, 1·57)	1·42 (1·29, 1·55)	-	-	-	-	-
Fibromyalgia/ Chronic Pain/Fatigue	1·38 (1·20, 1·60)	1·57 (1·38, 1·79)	1·42 (1·26, 1·60)	1·51 (1·33, 1·71)	1·56 (1·39, 1·75)	1·50 (1·34, 1·67)	1·36 (1·22, 1·52)	1·31 (1·17, 1·47)	1·44 (1·29, 1·61)	-	-	-	-	-
Depression	1·31 (1·14, 1·51)	1·41 (1·24, 1·60)	1·42 (1·26, 1·59)	1·44 (1·29, 1·61)	1·48 (1·33, 1·64)	1·42 (1·28, 1·57)	1·36 (1·24, 1·50)	1·36 (1·23, 1·50)	1·34 (1·22, 1·47)	-	-	-	-	-

ECI	1·22 (1·06, 1·41)	1·41 (1·24, 1·61)	1·41 (1·25, 1·59)	1·41 (1·26, 1·59)	1·48 (1·33, 1·65)	1·38 (1·24, 1·53)	1·33 (1·21, 1·48)	1·31 (1·19, 1·45)	1·31 (1·19, 1·44)	1·39 (1·27, 1·53)	1·47 (1·33, 1·63)	1·58 (1·43, 1·76)	1·55 (1·39, 1·72)	1·59 (1·43, 1·78)
Single ECI[a], urbanicity, and age	1·19 (1·01, 1·39)	1·35 (1·17, 1·59)	1·40 (1·23, 1·60)	1·32 (1·16, 1·50)	1·38 (1·23, 1·55)	1·30 (1·15, 1·46)	1·27 (1·13, 1·42)	1·25 (1·11, 1·40)	1·30 (1·17, 1·45)	1·33 (1·19, 1·48)	1·35 (1·21, 1·52)	1·55 (1·38, 1·75)	1·49 (1·32, 1·68)	1·47 (1·29, 1·67)
**≥1 incidents of total daily MMEs ≥ 90 for longer than 7 consecutive days**														
Adjusted for:														
Unadjusted	1·42 (1·24, 1·64)	1·51 (1·33, 1·71)	1·64 (1·45, 1·85)	2·00 (1·74, 29)	1·91 (1·68, 16)	1·77 (1·56, 2·00)	1·82 (1·63, 2·04)	1·82 (1·63, 2·03)	1·84 (1·65,2·04)	1·91 (1·71, 2·12)	1·97 (1·76, 2·19)	2·20 (1·96, 2·46)	2·05 (1·83, 2·29)	2·18 (1·94, 2·45)
Death in Cross-Sectional Year	1·39 (1·19, 1·61)	1·48 (1·29, 1·69)	1·47 (1·29, 1·68)	1·99 (1·69, 2·34)	1·87 (1·60, 2·18)	1·76 (1·51, 2·05)	1·84 (1·60, 2·12)	1·65 (1·44, 1·90)	1·69 (1·47, 1·94)	1·88 (1·69, 2·10)	1·96 (1·75, 2·19)	2·19 (1·95, 2·46)	2·06 (1·83, 2·31)	2·21 (1·96, 2·49)
Anxiety	1·40 (1·21, 1·61)	1·48 (1·30, 1·68)	1·59 (1·41, 1·80)	1·95 (1·69, 2·25)	1·82 (1·60, 2·07)	1·69 (1·48, 1·92)	1·77 (1·57, 1·99)	1·70 (1·52, 1·91)	1·74 (1·56, 1·94)	-	-	-	-	-
Fibromyalgia/ Chronic Pain/Fatigue	1·39 (1·19, 1·61)	1·48 (1·29, 1·69)	1·47 (1·29, 1·68)	1·99 (1·69, 2·34)	1·87 (1·60, 2·18)	1·76 (1·51, 2·05)	1·84 (1·60, 2·12)	1·65 (1·44, 1·90)	1·69 (1·47, 1·94)	-	-	-	-	-
Depression	1·33 (1·15, 1·54)	1·41 (1·24, 1·61)	1·53 (1·35, 1·73)	1·83 (1·58, 2·11)	1·71 (1·50, 1·95)	1·56 (1·37, 1·77)	1·68 (1·50, 1·89)	1·64 (1·46, 1·84)	1·65 (1·48, 1·84)	-	-	-	-	-
ECI	1·27 (1·09, 1·48)	1·37 (1·20, 1·57)	1·55 (1·36, 1·76)	1·83 (1·58, 2·12)	1·72 (1·50, 1·97)	1·52 (1·33, 1·73)	1·69 (1·50, 1·91)	1·62 (1·43, 1·83)	1·64 (1·46, 1·83)	1·75 (1·56, 1·96)	1·86 (1·66, 2·10)	2·04 (1·81, 2·31)	1·98 (1·75, 2·23)	2·22 (1·95, 2·53)
Single ECI[a], urbanicity, and age	1·27 (1·08, 1·50)	1·32 (1·14, 1·53)	1·55 (1·34, 1·79)	1·61 (1·36, 1·91)	1·46 (1·25, 1·71)	1·38 (1·18, 1·61)	1·67 (1·45, 1·92)	1·58 (1·37, 1·82)	1·54 (1·35, 1·76)	1·69 (1·48, 1·93)	1·69 (1·47, 1·95)	1·99 (1·72, 2·30)	1·99 (1·72, 2·31)	2·07 (1·77, 2·41)
**≥1 incidents of total daily MME ≥ 120 for longer than 7 consecutive days**														
Adjusted for:														
Unadjusted	1·53 (1·28, 1·82)	1·69 (1·45, 1·98)	1·91 (1·65, 2·21)	2·14 (1·83, 2·50)	1·99 (1·72, 2·30)	1·84 (1·60, 2·12)	1·90 (1·67, 2·17)	1·96 (1·72, 2·23)	1·92 (1·70, 2·17)	2·27 (2·00, 2·56)	2·19 (1·93, 2·49)	2·36 (2·07, 2·69)	2·30 (2·02, 2·62)	2·46 (2·15, 2·82)
Death in Cross-Sectional Year	1·49 (1·25, 1·77)	1·65 (1·41, 1·94)	1·85 (1·60, 2·15)	2·13 (1·82, 2·50)	1·98 (1·71, 2·29)	1·83 (1·58, 2·11)	1·86 (1·63, 2·13)	1·99 (1·74, 2·27)	1·88 (1·66, 2·13)	2·23 (1·97, 2·53)	2·18 (1·91, 2·48)	2·41 (2·11, 2·75)	2·32 (2·03, 2·65)	2·52 (2·19, 2·90)
Anxiety	1·50 (1·26, 1·79)	1·66 (1·42, 1·94)	1·87 (1·61, 16)	2·11 (1·80, 2·48)	1·91 (1·65, 2·21)	1·79 (1·54, 2·07)	1·86 (1·62, 2·13)	1·87 (1·63, 2·14)	1·82 (1·60, 2·06)	-	-	-	-	-
Fibromyalgia/ Chronic Pain/Fatigue	1·53 (1·27, 1·85)	1·76 (1·49, 2·08)	1·78 (1·51, 2·09)	2·09 (1·74, 2·51)	1·98 (1·65, 2·36)	1·89 (1·58, 2·26)	1·96 (1·66, 2·32)	1·79 (1·51, 2·13)	1·75 (1·49, 2·06)	-	-	-	-	-
Depression	1·41 (1·18, 1·69)	1·56 (1·33, 1·83)	1·77 (1·52, 2·05)	1·96 (1·67, 2·31)	1·80 (1·55, 2·09)	1·62 (1·40, 1·88)	1·75 (1·53, 2·01)	1·81 (1·58, 2·07)	1·72 (1·51, 1·95)	-	-	-	-	-

ECI	1·37 (1·14, 1·65)	1·55 (1·32, 1·84)	1·81 (1·55, 2·11)	1·98 (1·68, 2·34)	1·83 (1·57, 2·14)	1·61 (1·38, 1·87)	1·74 (1·51, 2·00)	1·81 (1·57, 2·09)	1·73 (1·52, 1·97)	2·12 (1·85, 2·42)	2·15 (1·88, 2·47)	2·25 (1·96, 2·60)	2·25 (1·96, 2·59)	2·49 (2·15, 2·88)
Single ECI[a], urbanicity, and age	1·39 (1·13, 1·72)	1·48 (1·23, 1·79)	1·86 (1·55, 2·22)	1·74 (1·43, 2·12)	1·60 (1·34, 1·92)	1·45 (1·21, 1·73)	1·73 (1·47, 2·04)	1·74 (1·47, 2·06)	1·56 (1·34, 1·82)	2·06 (1·76, 2·41)	1·91 (1·62, 2·25)	2·30 (1·94, 2·74)	2·32 (1·95, 2·75)	2·22 (1·86, 2·64)
**≥ 90 consecutive days of opioid coverage**														
Adjusted for:														
Unadjusted	1·52 (1·31, 1·78)	1·65 (1·44, 1·89)	1·58 (1·40, 1·79)	1·75 (1·56, 1·97)	1·58 (1·42, 1·76)	1·70 (1·54, 1·88)	1·54 (1·40, 1·69)	1·58 (1·44, 1·73)	1·56 (1·43, 1·70)	1·57 (1·44, 1·70)	1·52 (1·39, 1·65)	1·67 (1·54, 1·81)	1·61 (1·49, 1·74)	1·68 (1·55, 1·82)
Death in Cross-Sectional Year	1·56 (1·33, 1·82)	1·67 (1·46, 1·91)	1·59 (1·40, 1·80)	1·78 (1·58, 2·00)	1·59 (1·43, 1·77)	1·72 (1·55, 1·90)	1·55 (1·41, 1·71)	1·59 (1·45, 1·74)	1·57 (1·44, 1·72)	1·58 (1·45, 1·71)	1·53 (1·40, 1·66)	1·69 (1·56, 1·83)	1·61 (1·49, 1·74)	1·68 (1·56, 1·82)
Anxiety	1·48 (1·27, 1·73)	1·62 (1·41, 1·86)	1·54 (1·36, 1·75)	1·72 (1·53, 1·94)	1·52 (1·36, 1·69)	1·63 (1·47, 1·81)	1·49 (1·35, 1·64)	1·50 (1·36, 1·65)	1·47 (1·34, 1·61)	-	-	-	-	-
Fibromyalgia/ Chronic Pain/Fatigue	1·54 (1·31, 1·81)	1·69 (1·47, 1·95)	1·56 (1·36, 1·78)	1·72 (1·51, 1·95)	1·53 (1·36, 1·72)	1·57 (1·40, 1·76)	1·47 (1·32, 1·63)	1·43 (1·28, 1·59)	1·45 (1·31, 1·61)	-	-	-	-	-
Depression	1·43 (1·23, 1·68)	1·54 (1·34, 1·77)	1·51 (1·33, 1·71)	1·66 (1·48, 1·87)	1·48 (1·32, 1·64)	1·58 (1·42, 1·75)	1·46 (1·32, 1·60)	1·47 (1·34, 1·62)	1·45 (1·33, 1·59)	-	-	-	-	-
ECI	1·37 (1·17, 1·61)	1·58 (1·37, 1·82)	1·49 (1·31, 1·69)	1·65 (1·47, 1·87)	1·48 (1·33, 1·65)	1·55 (1·40, 1·72)	1·46 (1·33, 1·61)	1·44 (1·31, 1·59)	1·42 (1·29, 1·55)	1·47 (1·35, 1·60)	1·43 (1·31, 1·55)	1·57 (1·45, 1·71)	1·55 (1·43, 1·68)	1·61 (1·49, 1·75)
Single ECI[a], urbanicity, and age	1·38 (1·16, 1·64)	1·51 (1·29, 1·76)	1·53 (1·33, 1·76)	1·62 (1·42, 1·85)	1·33 (1·18, 1·50)	1·48 (1·32, 1·66)	1·40 (1·26, 1·56)	1·40 (1·26, 1·56)	1·41 (1·28, 1·57)	1·45 (1·31, 1·59)	1·41 (1·28, 1·56)	1·56 (1·42, 1·71)	1·52 (1·39, 1·67)	1·57 (1·43, 1·72)

[a] Single ECI models were adjusted for the conditions included in the index: alcohol abuse, chronic blood loss anemia, chronic pulmonary disease, coagulopathy, congestive heart failure, deficiency anemias, depression, diabetes (no complications), diabetes (chronic complications), drug abuse, fluid and electrolyte disorder, hypertension, hypothyroidism, liver disease, lymphoma, metastatic cancer, obesity, other neurological disorders, paralysis, peptic ulcer disease, peripheral vascular disease, psychoses, pulmonary circulatory disease, renal failure, rheumatoid arthritis, solid tumor (no metastasis), valvular disease, and weight loss.

Abbreviations: ECI: Elixhauser Comorbidity Index, HIV: human immunodeficiency virus, MME: morphine milligram equivalent, PWH: people living with HIV

Supplementary Table 8. Odds ratios of opioid prescriptions in PWH compared to HIV-negative stratified by sex (unadjusted), by calendar year

**Year**	2008	2009	2010	2011	2012	2013	2014	2015	2016	2017	2018	2019	2020	2021
**Outcome**	Odds ratio (95% CI)													
**≥1 opioid prescriptions in the last year**														
All	1·43 (1·33, 1·53)	1·40 (1·32, 1·50)	1·37 (1·30, 1·46)	1·38 (1·30, 1·46)	1·41 (1·34, 1·49)	1·35 (1·28, 1·42)	1·39 (1·32, 1·45)	1·37 (1·31, 1·43)	1·33 (1·27, 1·39)	1·40 (1·35, 1·46)	1·39 (1·33, 1·44)	1·41 (1·36, 1·47)	1·38 (1·33, 1·43)	1·46 (1·41, 1·51)
Men	1·45 (1·35, 1·58)	1·47 (1·36, 1·59)	1·44 (1·34, 1·55)	1·44 (1·35, 1·54)	1·49 (1·40, 1·59)	1·39 (1·31, 1·47)	1·45 (1·37, 1·53)	1·42 (1·35, 1·50)	1·34 (1·27, 1·40)	1·39 (1·33, 1·46)	1·37 (1·31, 1·43)	1·39 (1·33, 1·46)	1·39 (1·33, 1·45)	1·46 (1·40, 1·52)
Women	1·37 (1·22, 1·55)	1·27 (1·13, 1·42)	1·24 (1·12, 1·38)	1·25 (1·13, 1·38)	1·25 (1·14, 1·38)	1·25 (1·14, 1·37)	1·25 (1·15, 1·36)	1·25 (1·15, 1·36)	1·31 (1·21, 1·42)	1·43 (1·32, 1·54)	1·43 (1·33, 1·55)	1·47 (1·37, 1·58)	1·35 (1·25, 1·45)	1·46 (1·36, 1·57)

Abbreviations: HIV: human immunodeficiency virus, PWH: people with HIV

Supplementary Table 9. Odds ratios of any opioid use disorder (OUD) indicator in PWH compared to HIV-negative stratified by sex (unadjusted), by calendar year

Year	2008	2009	2010	2011	2012	2013	2014	2015	2016
**Outcome**	Odds ratio (95% CI)								
**Any OUD indicator**									
All	3·83 (2·85, 5·16)	3·98 (3·02, 5·24)	3·19 (2·55, 4·00)	3·33 (2·67, 4·14)	2·68 (2·21, 3·26)	2·95 (2·49, 3·51)	2·71 (2·34, 3·15)	2·62 (2·30, 2·99)	2·12 (1·90, 2·35)
Men	3·48 (2·48, 4·88)	4·09 (2·98, 5·53)	3·43 (2·64, 4·45)	3·48 (2·71, 4·49)	2·50 (2·00, 3·12)	2·99 (2·46, 3·64)	2·69 (2·27, 3·18)	2·62 (2·25, 3·05)	2·17 (1·92, 2·46)
Women	5·29 (2·81, 9·95)	3·65 (2·10, 6·34)	2·61 (1·68, 4·06)	2·89 (1·86, 4·49)	3·35 (2·26, 4·96)	2·83 (1·97, 4·06)	2·81 (2·06, 3·85)	2·63 (2·02, 2·43)	1·97 (1·61, 2·43)

Abbreviations: HIV: human immunodeficiency virus, PWH: people with HIV

Supplementary Table 10. Odds ratios of indicators of OUD in Medicare beneficiaries with HIV compared to matched HIV-negative beneficiaries, unadjusted and adjusted for anxiety, fibromyalgia/chronic pain/fatigue, depression, or ECI[a], by calendar year

**Year**	2008	2009	2010	2011	2012	2013	2014	2015	2016
**Outcome**	Odds ratio (95% CI)								
**Any OUD indicator**									
Adjusted for:									
Unadjusted	3·83 (2·85, 5·16)	3·98 (3·02, 5·24)	3·19 (2·55, 4·00)	3·33 (2·67, 4·14)	2·68 (2·21, 3·26)	2·95 (2·49, 3·51)	2·71 (2·34, 3·15)	2·62 (2·30, 2·99)	2·12 (1·90, 2·35)
Death in Cross-Sectional Year	3·72 (2·75, 5·02)	3·94 (2·99, 5·20)	3·17 (2·52, 3·98)	3·26 (2·61, 4·06)	2·64 (2·18, 3·21)	2·93 (2·47, 3·48)	2·64 (2·27, 3·07)	2·55 (2·23, 2·91)	2·10 (1·89, 2·34)
Anxiety	3·97 (2·88, 5·46))	3·51 (2·61, 4·71)	2·98 (2·35, 3·79)	3·42 (2·70, 4·32)	2·59 (2·11, 3·17)	2·77 (2·30, 3·34)	2·64 (2·25, 3·09)	2·36 (2·05, 2·72)	1·93 (1·72, 2·16)
Fibromyalgia/ Chronic Pain/Fatigue	4·33 (3·10, 6·08)	3·84 (2·87, 5·14)	3·01 (2·36, 3·84)	3·24 (2·54, 4·15)	2·67 (2·13, 3·33)	3·09 (2·52, 3·78)	2·72 (2·28, 3·25)	2·61 (2·22, 3·05)	2·13 (1·88, 2·42)
Depression	3·50 (2·58, 4·75)	3·48 (2·61, 4·63)	3·00 (2·37, 3·80)	3·11 (2·47, 3·91)	2·38 (1·94, 2·90)	2·55 (2·13, 3·07)	2·40 (2·05, 2·82)	2·25 (1·95, 2·59)	1·83 (1·63, 2·04)
ECI	3·33 (2·38, 4·65)	3·33 (2·47, 4·51)	3·04 (2·37, 3·90)	3·24 (2·51, 4·19)	2·22 (1·80, 2·75)	2·52 (2·07, 3·06)	2·33 (1·98, 2·75)	2·16 (1·86, 2·50)	1·76 (1·57, 1·99)
Single ECI[b], urbanicity, and age	3·23 (1·51, 6·90)	2·67 (1·62, 4·41)	2·61 (1·69, 4·03)	2·01 (1·30, 3·10)	1·27 (0·84, 1·92)	1·63 (1·14, 2·32)	1·58 (1·21, 2·07)	1·52 (1·17, 1·97)	1·15 (0·93, 1·41)
**Any OUD indicator – except MOUD**									
Unadjusted	3·63 (2·67, 4·95)	3·95 (2·95, 5·30)	3·02 (2·39, 3·83)	3·41 (2·70, 4·32)	2·65 (2·17, 3·25)	2·90 (2·42, 3·48)	2·58 (2·20, 3·02)	1·51 (2·19, 2·89)	1·99 (1·78, 2·22)
Death in Cross-Sectional Year	3·50 (2·56,4·78)	3·91 (2·91, 5·26)	2·98 (2·35, 3·79)	3·36 (2·65, 4·26)	2·61 (2·13, 3·20)	2·87 (2·39, 3·44)	2·51 (2·14, 2·94)	2·42 (2·10, 2·79)	1·97 (1·76, 2·21)
Anxiety	3·69 (2·65, 5·15)	3·42 (2·50, 4·69)	2·75 (2·15, 3·55)	3·53 (2·74, 4·56)	2·55 (2·06, 3·17)	2·72 (2·24, 3·31)	2·48 (2·10, 2·93)	2·26 (1·95, 2·62)	1·78 (1·58, 2·01)
Fibromyalgia/ Chronic Pain/Fatigue	4·15 (2·91, 5·93)	3·91 (2·85, 5·38)	2·86 (2·21, 3·70)	3·38 (2·59, 4·42)	2·69 (2·12, 3·41)	3·01 (2·42, 3·73)	2·64 (2·18, 3·19)	2·50 (2·12, 2·96)	1·96 (1·71, 2·24)
Depression	3·30 (2·41, 4·53)	3·37 (2·48, 4·58)	2·81 (2·19, 3·60)	3·10 (2·43, 3·96)	2·32 (1·88, 2·88)	2·52 (2·07, 3·06)	2·31 (1·95, 2·73)	2·18 (1·88, 2·52)	1·72 (1·52, 1·93)
ECI	3·10 (2·18, 4·39)	3·21 (2·33, 4·42)	2·91 (2·23, 3·79)	3·30 (2·50, 4·36)	2·18 (1·74, 2·73)	2·38 (1·93, 2·93)	2·23 (1·87, 2·66)	2·06 (1·77, 2·41)	1·61 (1·42, 1·83)
Single ECI[b], urbanicity, and age	2·34 (1·01, 5·41)	2·40 (1·38, 4·16)	2·43 (1·50, 3·92)	1·97 (1·21, 3·21)	1·32 (0·86, 2·05)	1·60 (1·11, 2·31)	1·53 (1·15, 2·04)	1·64 (1·25, 2·14)	1·02 (0·82, 1·27)
**Diagnosed OUD**									
Adjusted for:									

Unadjusted	4·22 (3·05, 5·84)	4·86 (3·35, 6·28)	3·67 (2·82, 4·76)	4·08 (3·18, 5·23)	2·82 (2·30, 3·46)	3·15 (2·62, 3·78)	2·75 (2·35, 3·22)	2·77 (2·40, 3·18)	2·22 (1·98, 2·50)
Death in Cross-Sectional Year	4·09 (2·95, 5·67)	4·52 (3·30, 6·20)	3·58 (2·74, 4·66)	4·00 (3·12, 5·15)	2·78 (2·27, 3·41)	3·12 (2·59, 3·75)	2·66 (2·27, 3·12)	2·68 (2·32, 3·09)	2·20 (1·95, 2·47)
Anxiety	4·37 (3·07, 6·21)	4·06 (2·87, 5·73)	3·50 (2·64, 4·64)	4·18 (3·19, 5·46)	2·68 (2·16, 3·33)	2·92 (2·40, 3·55)	2·66 (2·25, 3·14)	2·50 (2·15, 2·91)	2·05 (1·81, 2·32)
Fibromyalgia/ Chronic Pain/Fatigue	4·90 (3·37, 7·13)	4·73 (3·36, 6·67)	3·49 (2·62, 4·64)	4·00 (3·02, 5·29)	2·81 (2·22, 3·55)	3·31 (2·67, 4·10)	2·80 (2·32, 3·38)	2·85 (2·40, 3·38)	2·30 (2·00, 2·65)
Depression	3·90 (2·80, 5·43)	4·04 (2·92, 5·58)	3·55 (2·69, 4·69)	3·91 (3·01, 5·10)	2·46 (1·99, 3·05)	2·70 (2·22, 3·28)	2·43 (2·06, 2·87)	2·38 (2·05, 2·77)	1·92 (1·69, 2·17)
ECI	3·94 (2·70, 5·75)	4·08 (2·87, 5·79)	3·55 (2·64, 4·76)	4·10 (3·04, 5·54)	2·31 (1·85, 2·90)	2·64 (2·15, 3·25)	2·38 (2·00, 2·85)	2·28 (1·95, 2·67)	1·85 (1·62, 2·10)
Single ECI[b], urbanicity, and age	5·58 (1·82, 17·10)	3·20 (1·65, 6·20)	3·25 (1·68, 6·28)	2·61 (1·48, 4·60)	1·22 (0·75, 2·00)	1·62 (1·09, 2·41)	1·78 (1·31, 2·42)	1·81 (1·33, 2·46)	1·15 (0·88, 1·50)
**Opioid-related hospitalization**									
Adjusted for:									
Unadjusted	3·56 (2·56, 4·96)	3·69 (2·70, 5·05)	3·15 (2·45, 4·06)	3·24 (2·52, 4·17)	2·85 (2·27, 3·58)	3·50 (2·84, 4·30)	3·42 (2·82, 4·12)	3·28 (2·77, 3·89)	2·54 (2·22, 2·91)
Death in Cross-Sectional Year	3·35 (2·39, 4·69)	3·60 (2·63, 4·94)	3·14 (2·43, 4·06)	3·12 (2·41, 4·03)	2·78 (2·21, 3·50)	3·47 (2·82, 4·28)	3·21 (2·65, 3·90)	3·13 (2·63, 3·73)	2·60 (2·26, 2·98)
Anxiety	3·63 (2·53, 5·20)	3·11 (2·22, 4·36)	2·93 (2·34, 3·85)	3·35 (2·55, 4·41)	2·83 (2·21, 3·64)	3·23 (2·57, 4·05)	3·26 (2·66, 4·00)	3·03 (2·51, 3·66)	2·37 (2·04, 2·74)
Fibromyalgia/ Chronic Pain/Fatigue	4·22 (2·86, 6·22)	3·40 (2·44, 4·72)	3·03 (2·29, 4·00)	3·17 (2·38, 4·23)	2·76 (2·12, 3·59)	3·47 (2·72, 4·20)	3·30 (2·63, 4·13)	3·19 (2·61, 3·91)	2·57 (2·19, 3·02)
Depression	3·16 (2·24, 4·45)	2·99 (2·15, 4·16)	2·89 (2·21, 3·78)	2·88 (2·20, 3·77)	2·50 (1·97, 3·17)	3·03 (2·43, 3·78)	2·99 (2·44, 3·67)	2·79 (2·32, 3·35)	2·18 (1·89, 2·52)
ECI	2·98 (2·03, 4·36)	2·88 (2·03, 4·09)	3·10 (2·32, 4·16)	3·10 (2·82, 4·20)	2·25 (1·74, 92)	2·92 (2·27, 3·74)	2·74 (2·20, 3·42)	2·61 (2·13, 3·20)	2·08 (1·77, 2·45)
Single ECI[b], urbanicity, and age	3·10 (1·19, 8·06)	2·57 (1·40, 4·69)	1·96 (1·20, 3·21)	2·26 (1·36, 3·77)	1·60 (0·99, 2·57)	1·96 (1·28, 3·01)	1·60 (1·14, 2·24)	1·34 (0·96, 1·88)	1·20 (0·94, 1·54)
**Use of MOUD**									
Adjusted for:									
Unadjusted	7·50 (2·35, 23·91)	3·95 (1·81, 8·62)	4·20 (2·17, 8·15)	2·73 (1·49, 5·00)	2·86 (1·55, 5·27)	3·00 (1·85, 4·86)	3·61 (2·37, 5·52)	3·29 2· (21, 4·88)	3·38 (2·43, 4·71)
Death in Cross-Sectional Year	7·30 (2·29, 23·31)	4·03 (1·83, 8·88)	4·15 (2·14, 8·07)	2·61 (1·41, 4·82)	3·04 (1·62, 5·72)	3·01 (1·86, 4·89)	3·60 (2·35, 5·51)	3·29 (2·22, 4·89)	3·38 (2·43, 4·71)
Anxiety	9·72 (2·67, 35·37)	3·59 (1·60, 8·06)	5·43 (2·51, 11·76)	2·74 (1·49, 5·03)	2·59 (1·34, 4·99)	3·06 (1·82, 5·13)	3·74 (2·40, 5·84)	3·13 (2·05, 4·77)	3·30 (2·36, 4·61)

Fibromyalgia/ Chronic Pain/Fatigue	7·36 (2·30, 23·55)	3·83 (1·73, 8·47)	4·20 (2·05, 8·60)	2·59 (1·38, 4·86)	2·63 (1·36, 5·10)	3·21 (1·89, 5·45)	3·01 (1·92, 4·70)	3·25 (2·12, 4·99)	3·41 (2·42, 4·80)
Depression	10·86 (2·41, 48·93)	4·39 (1·84, 10·49)	4·52 (2·21, 9·24)	3·81 (1·85, 7·82)	3·17 (1·61, 6·24)	2·76 (1·66, 4·58)	2·93 (1·88, 4·58)	2·65 (1·68, 4·18)	2·90 (2·04, 4·11)
ECI	7·64 (2·10, 27·83)	3·75 (1·64, 8·57)	3·80 (1·93, 7·49)	2·81 (1·45, 5·45)	2·57 (1·34, 4·90)	3·03 (1·81, 5·08)	3·03 (1·94, 4·72)	2·90 (1·88, 4·47)	3·17 (2·26, 4·43)
Single ECI[b], urbanicity, and age	10·64 (1·35, 84·25)	9·50 (2·20; 41·13)	6·76 (2·07, 22·07)	2·65 (0·37, 19·24)	1·18 (0·11, 12·52)	-	1·68 (0·64, 4·39)	0·69 (0·18, 2·72)	4·95 (2·12, 11·54)

[a] Cells marked (-) were not calculable due to sample size.
[b] Single ECI models were adjusted for the conditions included in the index: alcohol abuse, chronic blood loss anemia, chronic pulmonary disease, coagulopathy, congestive heart failure, deficiency anemias, depression, diabetes (no complications), diabetes (chronic complications), drug abuse, fluid and electrolyte disorder, hypertension, hypothyroidism, liver disease, lymphoma, metastatic cancer, obesity, other neurological disorders, paralysis, peptic ulcer disease, peripheral vascular disease, psychoses, pulmonary circulatory disease, renal failure, rheumatoid arthritis, solid tumor (no metastasis), valvular disease, and weight loss.

Abbreviations: HIV: human immunodeficiency virus, MOUD: medication for OUD, OUD: opioid use disorder.
